# Speech and Deep Brain Stimulation in Parkinson's Disease, Essential Tremor, and Dystonia: A Systematic Review and Meta‐analysis

**DOI:** 10.1002/mds.70322

**Published:** 2026-04-30

**Authors:** Elina Tripoliti, Takashi Tsuboi, Michael T. Barbe, Mario Sousa, Julie Barkmeier‐Kraemer, Hannah Jergas, Gaia Arno, Nicole Buie, Mikayla Day, Petr Krýže, Jayson R. Nelson, Aaron E.L. Warren, Shervin Rahimpour, Jan Rusz, Paul Krack, Kristina Simonyan

**Affiliations:** ^1^ Department of Clinical and Movement Neurosciences UCL, Institute of Neurology and National Hospital for Neurology and Neurosurgery, UCLH NHS Trust London United Kingdom; ^2^ 1st Neurology Department National and Kapodistrian University of Athens, Medical School Athens Greece; ^3^ Department of Neurology Nagoya University Graduate School of Medicine Nagoya Japan; ^4^ Department of Neurology Faculty of Medicine and University Hospital Cologne, University of Cologne Cologne Germany; ^5^ Department of Neurology Inselspital University Hospital Bern Bern Switzerland; ^6^ Graduate School for Health Sciences University of Bern Bern Switzerland; ^7^ Department of Otolaryngology–Head and Neck Surgery University of Utah Salt Lake City Utah USA; ^8^ Department of Communication Sciences & Disorders University of Utah Salt Lake City Utah USA; ^9^ Department of Otolaryngology–Head & Neck Surgery Massachusetts Eye and Ear, Harvard Medical School Boston Massachusetts USA; ^10^ Department of Circuit Theory, Faculty of Electrical Engineering Czech Technical University in Prague Prague Czech Republic; ^11^ Department of Neurosurgery University of Utah, Clinical Neurosciences Center Salt Lake City Utah USA; ^12^ Department of Neurosurgery Mass General Brigham, Harvard Medical School Boston Massachusetts USA; ^13^ Department of Neurology Mass General Brigham Boston Massachusetts USA

**Keywords:** deep brain stimulation, dystonia, essential tremor, Parkinson's disease, speech

## Abstract

Deep brain stimulation (DBS) effectively treats motor symptoms in movement disorders but often compromises speech through incompletely defined mechanisms. We conducted a PROSPERO‐registered systematic review and meta‐analysis of publications through August 2024 (CRD42024527738). Among 2726 screened records, we included 184 studies: 131 in Parkinson's disease (PD), 32 in essential tremor (ET), and 21 in dystonia, assessing perceptual, acoustic, and patient‐reported speech outcomes. Meta‐analyses showed that subthalamic nucleus DBS in PD resulted in poorer speech intelligibility compared with best medical treatment (effect size −0.24; 95% confidence interval: −0.46 to −0.03; *P* = 0.027), with state‐dependent decline under active stimulation on longitudinal analysis (Unified Parkinson's Disease Rating Scale Part III item 18 monthly change: medication *on* +0.016, *P* < 0.001; medication *off* +0.002, *P* = 0.50). In ET, DBS consistently suppressed vocal tremor but increased the risk of dysarthria, particularly with bilateral stimulation. Dystonia outcomes showed greater heterogeneity. Across disorders, sustained phonation measures improved, whereas connected speech performance worsened, indicating selective vulnerability of complex motor tasks. Neuroanatomical mapping identified two nonexclusive mechanisms: current spread to corticobulbar fibers producing spastic speech features and to the cerebellothalamocortical pathway producing ataxic features. Hypokinetic and stuttering‐like phenotypes also occurred but likely reflect network‐level interactions rather than tract‐specific spread. These tract‐mediated and network‐level alterations appear to interact with hemispheric lateralization, medication state, and longer‐term plasticity to produce complex clinical phenotypes. We outline a clinical framework integrating systematic screening, phenotype identification, and targeted programming adjustments. Enhanced speech assessment, precise field mapping, and adaptive DBS paradigms may promote individualized care that optimizes speech and motor function in precision DBS therapy. © 2026 The Author(s). *Movement Disorders* published by Wiley Periodicals LLC on behalf of International Parkinson and Movement Disorder Society.

Parkinson's disease (PD), essential tremor (ET), and dystonia are among the most common movement disorders treated with deep brain stimulation (DBS).^1^ DBS effectively alleviates tremor and other motor symptoms, yet speech impairment represents one of its most frequent and functionally disabling side effects.^2^, ^3^, ^4^, ^5^, ^6^ DBS can enhance specific speech subsystems—particularly laryngeal control, as reflected in improved phonatory stability and loudness—yet worsen performance on connected speech, where articulatory precision and prosodic regulation are additionally required.^7^, ^8^, ^9^, ^10^, ^11^ This dissociation mirrors limb motor findings with subthalamic nucleus (STN)‐DBS, where rigidity and bradykinesia improve, yet performance on complex movement, including gait and balance, can deteriorate.^12^

Speech outcomes vary across disorders and stimulation targets. In PD, STN and globus pallidus internus (GPi) stimulation frequently induces dysarthria.^2^, ^3^, ^13^, ^14^, ^15^ In ET, thalamic ventral intermediate nucleus (Vim) or posterior subthalamic area/caudal zona incerta (PSA/cZi) stimulation typically suppresses voice tremor but may compromise intelligibility.^16^, ^17^, ^18^ In dystonia, GPi stimulation yields variable effects, ranging from perceptual improvement to newly emergent speech deficits.^19^, ^20^, ^21^ These heterogeneous outcomes likely result from interactions among disease pathophysiology, stimulation parameters, and stimulation‐induced network changes rather than a single pathway mechanism.^15^, ^22^

Despite these disorder‐specific observations, their cross‐disease synthesis remains scarce. Prior reviews have focused on single disorders or outcome modalities, and few have integrated perceptual, acoustic, and anatomical data.^23^, ^24^, ^25^, ^26^ To address these gaps, we conducted a comprehensive systematic review and meta‐analysis of speech outcomes after DBS in PD, ET, and dystonia. We classified studies by disease, target, and outcome type, integrating perceptual and acoustic findings with stimulation parameters and electrode localization. We aimed to characterize common and disorder‐specific patterns of speech modulation and to develop an evidence‐based, speech‐conscious programming framework. Cognitive‐linguistic aspects of communication, such as verbal fluency and word‐finding ability, were beyond the scope of this review. Furthermore, we provide insights that guide future approaches to enhance speech function in patients treated with DBS.

## Methods

### Protocol and Registration

This systematic review and meta‐analysis was conducted in accordance with the Preferred Reporting Items for Systematic reviews and Meta‐analysis (PRISMA) guidelines and was registered with PROSPERO (CRD42024527738).

### Eligibility Criteria (PICOS Framework)

The eligibility criteria for this review are:

- *Population:* patients diagnosed with PD, ET, or dystonia
- *Intervention:* DBS targeting STN, GPi, Vim, PSA/cZi, pedunculopontine nucleus (PPN), or related structures
- *Comparison:* studies with or without explicit comparison (eg, pre/post, ON/OFF, or between groups) were eligible if DBS‐related speech or voice outcomes were reported
- *Outcomes:* speech and/or voice changes measured by perceptual ratings, acoustic analyses, or patient‐reported outcomes (PROs)
- *Study design:* peer‐reviewed studies including four or more patients; case reports, conference abstracts, reviews, and non‐English publications were excluded

### Search Strategy and Data Extraction

A comprehensive literature search was conducted using PubMed, Embase, and PsycInfo databases via the Ovid interface up to September 2024. Search strings combined terms related to PD, ET, dystonia, speech or voice, and DBS (Supporting Information Note S1). Study screening and selection were managed using Covidence (https://www.covidence.org). After duplicate removal, three reviewers independently screened titles, abstracts, and full texts, resolving discrepancies by consensus with a fourth reviewer. Reference lists of included articles were manually checked for additional studies.

For each eligible study, data were extracted on study design, sample size, patient and control characteristics, DBS target and laterality, stimulation parameters, speech/voice outcome (perceptual, acoustic, or PROs), and electrode positions.

### Surgical Data

Studies reporting stereotactic coordinates of active contacts were included for further analysis.^27^, ^28^ Coordinates were reported either in the anterior commissure–posterior commissure (AC–PC) space or in the Montreal Neurological Institute (MNI) template space.^29^, ^30^ When only AC–PC coordinates were available, they were transformed into MNI space using the standard pipeline implemented in “LEAD DBS”.^31^

### Data Synthesis

Because of substantial methodological and outcome heterogeneity, most data were not statistically pooled. These studies were summarized descriptively, stratified by disorder, outcome domain, target, and study design. Where quantitative synthesis was feasible, meta‐analyses were performed as detailed in the following section.

### Meta‐Analyses

We conducted two separate meta‐analyses where statistical pooling was feasible. (Supporting Information Note S2). The first meta‐analysis used speech‐specific intelligibility outcomes to compare DBS and medically treated groups, although such data were limited. The second relied on Unified Parkinson's Disease Rating Scale Part III (UPDRS‐III) item 18, which, despite not being speech‐specific, was the only outcome measure consistently reported across studies comparing stimulation states. Specifically, the analyses invloved the following data:

1. STN‐DBS versus best medical treatment (between‐group, longitudinal): Prospective studies directly comparing PD cohorts treated with STN‐DBS vs. medically treated PD controls using speech intelligibility outcomes were included. Effect sizes were expressed as standardized mean differences (Hedges' *g*), where positive values indicate greater improvement in the DBS group compared with controls.
2. STN‐DBS (within‐group, longitudinal): We included studies that reported UPDRS‐III item 18 before and after STN‐DBS in PD in either medication *on* (Med‐*on*) or medication *off* (Med‐*off*), or both. Raw mean differences (Δ = follow‐up – baseline) were used, where positive values indicate worsening. Pooled estimates were synthesized within follow‐up bins (0.5–1 year, 2–4 years, >5 years).

For both analyses, when Δ values were unavailable, they were derived from baseline and follow‐up means and standard deviations (SDs), assuming a pre–post correlation of 0.5; sensitivity analyses used *r* = 0.3 and 0.7. Pooled estimates were calculated under a random‐effects model with restricted maximum likelihood. Heterogeneity was quantified using τ^2^, I^2^, and H^2^ statistics. Sensitivity analyses included leave‐one‐out tests (DBS vs. control), fixed‐ vs. random‐effects comparisons, and variation of correlation assumptions. Publication bias was assessed using funnel plots and Egger's regression test. All analyses were conducted in R (version 4.4.3) using the metafor package (version 4.8.0).

### Quality Assessment of the Included Studies

Risk of bias was evaluated using an adapted version of the PD‐specific tool by den Brok et al,^32^ modified to address speech outcomes. Results are presented in Tables S1A–C.

## Results

### Selection of Studies and Study Characteristics

The literature search identified 2726 records, of which 184 met the eligibility criteria after screening (131 PD, 32 ET, 21 dystonia; Fig. S1). Most were observational (cross‐sectional and longitudinal up to >10 years). Outcomes included perceptual rating, acoustic analyses, and PROs. Tables 1, 2, 3 summarize design, DBS targets, patient characteristics, and key findings for PD, ET, and dystonia studies, respectively.

**Table 1:** Studies reporting speech‐related outcomes for patients with PD

Authors	Study design	DBS target	No. of patients	Disease duration (yrs)	Age at assessment (yrs)	Control group	Summary of key results
Abeyesekera et al^72^	Prospective FU, 6 months	STN	10	Mean 10.6 [6–17]	Mean 63.9 [52–69]	NA	Voice quality improved postoperative Med‐On/STIM‐ON compared with preoperative Med‐On, with reduced jitter and shimmer and altered acoustic parameters for /a/, /o/, and /i/; no sex differences.
Aviles‐Olmos et al^162^	Prospective FU, 8 years	STN	41	12.9 ± 5.8	56.2 ± 8.4	NA	STN‐DBS improved motor and ADL outcomes long term, but communication declined significantly by 5 and 8 years.
Catalano Chiuve et al^116^	Prospective FU, 1 years	STN	14	8.2 ± 3.9	58.8 ± 7.9	PD‐Med HCs	STN‐DBS improved motor symptoms without worsening speech; PD‐BMT showed greater decline in vowel articulation over 12 months.
Dayal et al^90^	Prospective FU, 4 weeks	STN	16	20.4 ± 6.6	65.4 ± 6.4	NA	Short pulse width (30 μs) was as safe and effective as conventional settings, with no significant speech or clinical differences.
Dromey et al^181^	Prospective FU, 6 months	STN	7	NA	63.4 ± 12.8	NA	DBS improved limb motor function in PD, with minor increases in vocal intensity and pitch variability, but no meaningful speech changes.
Fabbri et al^9^	Prospective FU, 6 months	STN	10	12.1 ± 5.2	65.3 ± 6.1	PD‐Med	Low‐frequency stimulation improved speech intelligibility in patients with severe dysarthria, although motor benefits may diminish over time.
Fasano et al^35^	Prospective FU, 8 years	STN	20	13.7 ± 4.8	56.9 ± 7.2	NA	At 8 years post‐DBS, speech function worsened despite sustained motor improvement.
Frost et al^61^	Prospective FU, 6 months	STN	23	NA	Mean 58 [35–69]	PD‐Med	STN‐DBS led to variable speech outcomes; VHI scores correlated with intelligibility and declined similarly in surgical and medical groups.
Karl et al^82^	Prospective FU, 4 weeks	STN	20	19.3 ± 7.6	63.9 ± 6.1	NA	Dual‐frequency DBS improved gait, appendicular symptoms, speech ratings, and QoL compared with standard DBS settings.
Kluin et al^57^	Prospective FU, 1 month	STN	43	9.4 ± 5.2	64 ± 6.9	NA	Motor speech disorders affect 70%–90% post‐STN‐DBS; medial left lead placement and disease duration predict risk.
Knowles et al^83^	Prospective FU, 6 months	STN	12	10.7 ± 3.9	62.6 ± 6.0	NA	Low frequency (60 Hz) improved speech intelligibility and vowel articulation; outcomes varied individually and interacted with sex.
Krack et al^36^	Prospective FU, 5 years	STN	49	14.6 ± 5.0	55.0 ± 7.5	NA	Motor and ADL improvements persisted at 5 years post‐STN‐DBS, although speech and postural stability worsened *on* medication.
Martel Sauvageau et al^66^	Prospective FU, 1 year	STN	7	11.1 ± 2.8	65.9 ± 5.1	NA	STN‐DBS reduced vowel articulation *off* medication but improved it *on* medication.
Maruska et al^182^	Prospective FU, 3 months	STN	4	[11–19]	[41–54]	NA	Speech intelligibility generally improved with combined Med‐*on*/STIM‐ON conditions. Speaking rate, pitch, and loudness showed no consistent relationship with intelligibility.
Merola et al^60^	Prospective FU, 7 years	STN	19	22.8 ± 2.3	61.5 ± 5.7	NA	Speech difficulties increased from 16% pre‐DBS to 63% after >7 years, reflecting axial symptom progression and reduced dopaminergic responsiveness rather than stimulation‐specific effects.
Ostergaard et al^37^	Prospective FU, 4 years	STN	26	19 ± 5	59 ± 8	NA	Speech worsened significantly from 1 to 4 years post‐DBS, regardless of stimulation or medication state, reflecting progression of l‐dopa–resistant axial symptoms.
Paek et al^110^	Prospective FU, 6 months	STN	53	Mean 12 [5–31]	59.4 [27–73]	NA	Med‐*off* speech improved after STN‐DBS only when electrodes were accurately placed bilaterally in the STN, highlighting the importance of precise targeting.
Paek et al^38^	Prospective FU, 1 year	STN	57	12.6 ± 5.1	60.1 ± 8.7	NA	Accurate bilateral implantations led to significant speech improvement post‐DBS, whereas medial misplacement increased reversible speech deterioration.
Petry‐Schmelzer et al^91^	Prospective FU, 4 weeks	STN	24	8.8 ± 3.8	61.4 ± 8.7	NA	No significant differences in speech outcomes were found between 30 and 60 μs STN‐DBS, suggesting limited long‐term benefit of short pulse width for dysarthria.
Pinto et al^6^	Prospective FU, 2 years	STN	99	7.2 ± 2.9	52.4 ± 6.9	PD‐Med	Over 2 years, STN‐DBS did not significantly affect intelligibility or MPT vs. medical therapy; mild worsening in diadochokinesis and clinician‐rated speech occurred but was not perceived by patients.
Rousseaux et al^40^	Prospective FU, 3 months	STN	7	12.0 ± 2.2	61.7 ± 6.5	NA	STN‐DBS showed modest improvement in lip mobility and voice modulation, but intelligibility worsened in some patients, particularly when combined with l‐dopa, likely because of facial dyskinesia.
Sarac et al^183^	Prospective FU, 6 months	STN	12	NA	58.8 ± 9.7	NA	Females showed acoustic voice deterioration (reduced F0, increased jitter/shimmer) without perceptible changes on GRBAS; males showed no significant acoustic or perceptual changes.
Spielman et al^65^	Prospective FU, 6 months	STN	4	NA	[48–84]	PD‐Med	LSVT LOUD improved vocal loudness and vowel articulation with and without STN‐DBS at 6 months, although long‐term self‐perceived voice improvements were less consistent in the DBS group.
Tanaka et al^59^	Prospective FU, 2 years	STN	25	11.8 ± 4.1	65.0 ± 8.8	NA	Most patients showed worsened speech intelligibility and naturalness with STN‐DBS, while a minority improved in volume‐related features; poorer preoperative cognition predicted deterioration.
Tandra et al^41^	Prospective FU, 6 months	STN	40	Mean 7.3 [4–18]	Mean 55.5 [32–74]	NA	STN‐DBS improved speech scores in the Med‐*off* state, but not in the Med‐*on* state, suggesting limited dopaminergic responsiveness.
Thies et al^10^	Prospective FU, 9 months	STN	13	8 ± 5	58.9 ± 8.5	HCs	STN DBS improved speech intelligibility and loudness by enhancing lower lip kinematics, while tongue movements remained impaired, suggesting differential articulator response.
Tripoliti et al^184^	Prospective FU, 1 year	STN	8	NA	58	PD‐Med	STN‐DBS did not significantly alter sentence intelligibility overall, although two patients showed marked declines; increased SPL, jitter, and shimmer suggested possible voice quality deterioration.
Tripoliti et al^2^	Prospective FU, 3 years	STN	32	12.5 ± 4.7	58.8 ± 6.3	PD‐Med	STN‐DBS led to significant speech intelligibility decline in 78% of patients, despite increased loudness; poorer outcomes were linked to high left‐side voltage and medial or posterolateral electrode placement.
Tripoliti et al^114^	Prospective FU, 6 months	STN	10	13.6 ± 5.3	59.4 ± 4.5	PD‐Med	LSVT improved vocal loudness and perceptual ratings only in medically treated patients; in the STN‐DBS group, responses were variable with some deterioration, particularly in voice quality and respiratory support.
Tripoliti et al^7^	Prospective FU, 1 year	STN	54	12.5 ± 4.7	58.8 ± 6.3	NA	One year after STN‐DBS, speech intelligibility declined by ~14%, with articulation most affected; poorer preoperative speech, longer disease duration, and medial left electrode placement predicted greater deterioration.
Tsuboi et al^3^	Prospective FU, 1 year	STN	32	11.4	63.3 ± 9.1	PD‐Med	At baseline, ~90% patients had speech/voice disorders. After STN‐DBS, speech intelligibility and naturalness worsened, with strained voice (28%) and spastic dysarthria (44%) emerging as DBS‐induced disorders.
Wang et al^185^	Prospective FU, 3 months	STN	6	14.3 ± 2.3	55 ± 9	NA	Unilateral STN‐DBS showed mild respiratory/phonatory benefit only with right‐sided stimulation; left‐STN stimulation reduced vocal intensity and duration. Fundamental frequency remained unchanged.
Wang et al^113^	Prospective FU, 6 months	STN	20	12.3 ± 6.0	56.2 ± 10.6	NA	STN‐DBS affected speech in a hemisphere‐specific manner: left‐side stimulation reduced articulatory accuracy and speaking rate, whereas right‐side stimulation preserved or improved them.
Yasar et al^186^	Prospective FU, 1 month	STN	20	NA	60.2 ± 6.9	NA	STN‐DBS acutely improved voice quality in PD, reducing jitter, shimmer, and HNR, and enhancing HNR; DDK rates also improved, indicating better articulatory speed.
Rodriguez‐Oroz et al^39^	Prospective FU, 4 years	STN/GPi	STN: 49 GPi: 20	STN: 15.4 ± 6.3 GPi: 15.4 ± 6.3	STN: 59.8 ± 9.8 GPi: 55.8 ± 9.4	NA	Over 3–4 years, speech did not improve and often worsened in both STN and GPi groups; severe speech‐related adverse events were more frequent in the STN‐DBS group.
Pinto et al^107^	Prospective FU, 1 year	STN/PPN	7	21.7 ± 6.5	63.0 ± 5.8	NA	PPN stimulation impaired speech (MPT, DDK, intelligibility). l‐Dopa lessened STN speech benefits, with overall postoperative intelligibility decline.
Sidiropoulos et al^88^	Prospective FU, 3 months	STN/GPi/PPN	45	17.8 ± 5.7	59.5 ± 7.8	NA	No significant group‐level speech improvement was found between HFS and LFS, but 40% of patients reported subjective benefit; LFS may help alleviate HFS‐related speech impairment.
Eklund et al^187^	Prospective FU, 1 years	STN/cZi	19	STN: 7.2 ± 2.1 cZi: 6.3 ± 2.7	STN: 61.8 cZi: 60.4	NA	cZi‐DBS significantly worsened speech articulation compared with STN‐DBS.
Karlsson et al^188^	Prospective FU, 1 year	STN/cZi	STN: 7 cZi: 7	STN: 6.4 ± 1.5 cZi: 5.6 ± 2.5	STN: 62.2 ± 8.2 cZi: 61.9 ± 9.0	NA	STN‐DBS increased speech rate without compromising quality, whereas cZi‐DBS significantly reduced both speech rate and quality.
Karlsson et al^189^	Prospective FU, 1 year	STN/cZi	STN: 8 cZi: 8	STN: 6.8 ± 1.7 cZi: 6.1 ± 2.8	STN: 63.0 ± 7.9 cZi: 60.8 ± 9.0	NA	Phonatory control declined progressively in PD, with increased inappropriate voicing postoperatively; STN‐ and cZi‐DBS had no significant effect.
Karlsson et al^106^	Prospective FU, 1 year	STN/cZi	STN: 8 cZi: 8	STN: 6.8 ± 1.7 cZi: 6.1 ± 2.8	STN: 63.0 ± 7.9 cZi: 60.8 ± 9.0	NA	STN‐DBS increased pitch variation and range, whereas cZi‐DBS had no significant effect; cZi‐DBS patients showed postsurgical decline in pitch dynamics.
Lundgren et al^74^	Prospective FU, 1 year	STN/cZi	STN: 8 cZi: 8	STN: 6.8 ± 1.7 cZi: 6.1 ± 2.8	STN: 62.6 ± 7.8 cZi: 60.3 ± 8.9	NA	STN‐DBS increased voice intensity during reading, whereas cZi‐DBS decreased it.
Mate et al^99^	Prospective FU, 1 month	STN/GPi/Vim	STN: 23 GPi: 2 Vim: 2	13.7 ± 4.8	61 ± 7.7	NA	DBS and l‐dopa improved sustained vowel phonation in PD, notably reducing jitter; subjective voice quality also improved, although some acoustic gains lacked statistical significance.
Johansson et al^104^	Prospective FU, 1 year	cZi	10	6.3 ± 2.7	60.4 ± 8.7	NA	cZi‐DBS significantly reduced word‐level speech intelligibility, with further deterioration under noise.
Sandstrom et al^105^	Prospective FU, 1 year	cZi	11	7.1 ± 2.8	57.3 ± 8.7	HCs	At 6 months, cZi‐DBS reduced spontaneous speech intelligibility in most patients, although effects diminished by 12 months; l‐dopa significantly improved intelligibility.
Cavallieri et al^78^	Retrospective longitudinal FU, 5 years	STN	25	10.4 ± 4.6	58.4 ± 5.7	NA	Louder speech correlated with greater gait acceleration, whereas poorer voice quality and slower speech rate were linked to impaired gait and balance.
El Ouadih et al^42^	Retrospective longitudinal FU, 1 year	STN	92	10.4 ± 3.8	60.9 ± 6.9	NA	Symmetric STN‐DBS contact settings were strongly linked to speech deterioration in PD, reducing UPDRS speech scores and blunting dopaminergic benefits.
Gessani et al^43^	Retrospective longitudinal FU, 5 years	STN	25	10.4 ± 4.6	58.4 ± 5.7	NA	STN‐DBS acutely improved speech intelligibility, voice intensity, and phonation time, but long‐term effects varied.
Jorge et al^44^	Retrospective longitudinal FU, 6 months	STN	14	NA	72 ± 5	NA	Voice improvements colocalized with anterior sensorimotor left STN stimulation.
Levy et al^69^	Retrospective longitudinal FU, 16 months	STN	48	10.5 ± 4.3	58 ± 8	NA	Speech intelligibility declined after STN‐DBS, whereas acoustic parameters remained stable, and intelligibility changes showed weak correlation with MER data and varied by gender.
Li et al^45^	Retrospective longitudinal FU, 5 years	STN	195	6.8 ± 4.2	58.2 ± 10.0	NA	STN‐DBS improved *off*‐medication speech at 1–3 years but returned to baseline by year 5; *on*‐medication speech declined significantly.
Shahidi et al^46^	Retrospective longitudinal FU, 6 years	STN	37	11.3 ± 1.9	50 ± 3	NA	Speech scores on UPDRS‐III significantly worsened 6 years after STN‐DBS despite initial postoperative stability at 6 months.
Vijiaratnam et al^190^	Retrospective longitudinal FU, 36 months	STN	LFS: 20 HFS: 96	LFS: 12.8 ± 5.0 HFS: 12.4 ± 5.6	LFS: 59.3 ± 8.4 HFS: 56.5 ± 9.4	NA	LFS improved speech intelligibility and communication‐related QoL, especially in the Med‐*on* state, likely by enhancing phonation‐respiration coordination through selective modulation of brain oscillations.
Zibetti et al^86^	Retrospective longitudinal FU, 2 years	STN	85	12.8 ± 4.9	58.5 ± 8.1	NA	LFS led to subjective speech improvement in many patients, especially those with prior HFS‐related speech issues; although short‐term objective gains were limited, LFS slowed long‐term speech deterioration.
Hariz et al^103^	Retrospective longitudinal FU, 4 years	STN/GPi	STN: 49 GPi: 20	STN: 15.4 ± 6.3 GPi: 15.4 ± 6.2	STN: 62.9 ± 9.6 GPi: 59.8 ± 9.5	NA	Speech‐related adverse events, including dysphonia and dysarthria, were observed at 4 years postsurgery, more frequently and severely in STN‐DBS than in GPi‐DBS.
Patel et al^100^	Retrospective	STN/GPi/Vim	510 (PD, ET, dystonia)	NA	59.1 ± 14.3	NA	Speech disturbance occurred in 3.1% of cases post‐DBS; disease progression and medical therapy may be confounders.
Picillo et al^62^	Retrospective	STN/GPi	453	Median 13	Median 60	NA	Stuttering developed in 3.5% of patients post‐DBS, often with familial history; effects occurred after both STN and GPi‐DBS, suggesting genetic and disease‐related vulnerability.
Chiu et al^96^	Retrospective longitudinal FU, 29 months	GPi	25	12.5	63.7 ± 7.6	NA	Bilateral GPi‐DBS worsened multiple speech subsystems without short‐term intelligibility loss, although dysarthrias emerged and long‐term decline was suggested.
Tsuboi et al^101^	Retrospective longitudinal FU, 6 years	GPi	65	11.9 ± 5.4	62.6 ± 9.2	PD‐Med	GPi‐DBS did not significantly change speech in the Med‐*off* state, but Med‐*on* speech worsened significantly from 3 years postsurgery, indicating disease progression.
Hariz et al^97^	Retrospective longitudinal FU, 6.6 years	Vim	38	8.27 ± 3.8	63.7 ± 11.6	NA	Axial symptoms, including speech, worsened at 6 years post‐Vim‐DBS regardless of stimulation.
Ahlberg et al^71^	Cross‐sectional	STN	4	24.0 ± 9.9	71.0 ± 7.5	NA	STN‐DBS led to communication difficulties despite improved QoL; participants emphasized compensatory strategies and the need for support and information on speech‐related side effects.
Ahn et al^191^	Cross‐sectional	STN	7	Mean 12.3	Mean 57	NA	STN‐DBS altered spontaneous speech by shortening and increasing long pauses, raising nonlinguistic boundary pauses, and potentially impairing lexical retrieval and speech fluency.
Astrom et al^94^	Cross‐sectional	STN	10	NA	59 ± 7	NA	High‐amplitude STN‐DBS improved motor function but impaired speech intelligibility in some patients, especially when stimulation affected the fasciculus cerebellothalamicus.
Atkinson‐Clement et al^177^	Cross‐sectional	STN	11	10.5 ± 3.2	59.6 ± 6.3	NA	STN‐DBS moderately improved speech intelligibility during specific tasks, although effects varied and lacked consistent neural correlates.
Atkinson‐Clement et al^192^	Cross‐sectional	STN	12	NA	60.8 ± 8.1	NA	Combined STN‐DBS and l‐dopa treatment impaired vowel production, suggesting a negative interaction impacting speech‐related motor control.
Avantaggiato et al^193^	Cross‐sectional	STN	9	[5–17]	NA	NA	Left STN beta‐low power is associated with overt speech production, whereas right STN beta‐high power correlates with speech intelligibility and articulatory precision.
Behroozmand et al^194^	Cross‐sectional	STN	10	11.0 ± 3.0	64.8 ± 7.1	NA	DBS reduced voice jitter but did not alter pitch compensation or intelligibility, likely because of dysarthrogenic effects on articulation.
Bobin et al^22^	Cross‐sectional	STN	38	[34–77]	62,5 ± 8.5	NA	STN‐DBS improved speech/voice depending on stimulation laterality, intensity, and location; right STN stimulation and low‐to‐medium intensities yielded the most consistent benefits.
D'Alatri et al^195^	Cross‐sectional	STN	12	12.5 ± 5.7	60.3 ± 7.5	NA	STN‐DBS improved motor function and vocal tremor stability without worsening speech, although effects on intelligibility and overall dysarthria were limited.
Dayal et al^89^	Cross‐sectional	STN	15	14 ± 4	55 ± 8	NA	Short pulse width (30 μs) widened the therapeutic window and improved gait and speech intelligibility compared with standard pulse width (60 μs).
Dromey et al^55^	Cross‐sectional	STN	6	13.8 ± 6.3	59.8 ± 11.6	NA	STN‐DBS produced variable speech outcomes in PD; most showed poorer perceptual ratings and verbal fluency.
Duez et al^196^	Cross‐sectional	STN	10	14.2 ± 5.0	62.2 ± 8.7	HCs	STN‐DBS preserved prosodic syntax; F₀ differed between controls and STIM‐OFF patients, whereas articulation rate and pause measures showed no significant group differences.
Fabbri et al^92^	Cross‐sectional	STN	7	11.1 ± 3.1	56.2 ± 8.2	NA	30‐μs stimulation improved speech intelligibility without correlating with motor gains; side effects included dysarthria and facial contractions.
Fenoy et al^197^	Cross‐sectional	STN	35	AR: 9.6 ± 3.1 TD: 8.2 ± 3.7	AR: 66 ± 8 TD: 60 ± 8	NA	STN‐DBS worsened speech more in AR‐PD than TD‐PD, particularly with left medial contacts involving the dentatorubrothalamic tract.
Gentil et al^8^	Cross‐sectional	STN	10	11 ± 4	49 ± 9	HCs	STN‐DBS improved motor function and articulatory control, enhancing strength, accuracy, and timing of speech organs.
Gentil et al^198^	Cross‐sectional	STN	26	14 ± 4	55 ± 7	NA	STN‐DBS altered phonatory features, increasing vowel duration, shortening sentence duration, and affecting f₀ variability.
Gentil et al^199^	Cross‐sectional	STN	16	18 ± 6	54 ± 8	NA	STN‐DBS improved articulation speed, strength, and precision; enhanced phonatory and respiratory functions; and extended phonation time.
Grover et al^80^	Cross‐sectional	STN	15	10.6 ± 3.8	65.0 ± 5.8	NA	LFS improved speech intelligibility and verbal fluency switching, whereas HFS impaired multiple speech domains.
Haarmann et al^63^	Cross‐sectional	STN	186	11.6 ± 5.5	67.3 ± 8.9	PD‐Med	PD‐DBS patients showed greater impairment in speech, gait, and posture than PD‐Med patients.
Hammer et al^112^	Cross‐sectional	STN	18	Mean 11.5	59.6 ± 13.5	NA	STN‐DBS altered laryngeal and respiratory control, causing overdrive; effects correlated with stimulation parameters, favoring lower‐frequency settings for speech.
Hammer et al^81^	Cross‐sectional	STN	17	Mean 12	59.2 ± 13.8	NA	STN‐DBS subtly altered velopharyngeal control, increasing intraoral pressure in some; effects varied and favored low‐frequency stimulation.
Jergas et al^70^	Cross‐sectional	STN	25	NA	65.1 ± 8.3	NA	Stimulation‐induced dysarthria involves lateral and posteromedial fiber pathways. Both hemispheres showed similar susceptibility patterns.
Klostermann et al^48^	Cross‐sectional	STN	19	NA	NA	NA	STN‐DBS worsened perceptual speech ratings despite gains in specific acoustic parameters, with dysarthrogenic effects overshadowing motor benefits.
Knowles et al^200^	Cross‐sectional	STN	12	13.8 ± 5.0	64.3 ± 6.0	PD‐Med HCs	Patients with DBS showed greater baseline impairment and smaller acoustic gains at faster speaking rates.
Kryze et al^201^	Cross‐sectional	STN	32	17.5 ± 4.3	61.2 ± 6.9	HCs	A minimum 128‐word passage is required for stable acoustic assessment post‐STN‐DBS in PD.
Lange et al^15^	Cross‐sectional	STN	24	[4–30]	[50–81]	NA	Spastic and strained types of stimulation‐induced dysarthria tended to worsen under STN‐DBS, each associated with distinct patterns of connectivity.
Lee et al^202^	Cross‐sectional	STN	19	9.3	63.8	PD‐Med HCs	DBS reduced correlation dimension values in patients with PD compared with PD‐Med and HCs, indicating improved voice quality.
Little et al^95^	Cross‐sectional	STN	8	NA	NA	NA	Adaptive DBS showed lower speech side effects and improved motor function with reduced thresholds.
Lizarraga et al^93^	Cross‐sectional	STN	22	17	Mean 65.5 (range, 51–79)	NA	Lateralized STN‐DBS in PD showed no significant changes in speech parameters, including pitch, loudness, jitter, and shimmer.
Mahlknecht et al^14^	Cross‐sectional	STN	21	Median 11.3 [7.2–13.8]	Median 60.6 [52.6–67.9]	NA	Post‐STN‐DBS “lateral‐type” speech impairment was linked to pyramidal tract activation, whereas “medial‐type” speech was unrelated to pyramidal activation.
Martel Sauvageau et al^66^	Cross‐sectional	STN	8	[9–25]	[53–72]	NA	STN‐DBS improved vowel articulation by expanding vowel space and reducing formant centralization, although overall intelligibility gains remained limited.
Martel‐Sauvageau 2017^77^	Cross‐sectional	STN	8	[9–25]	[53–72]	HCs	STN‐DBS altered vocalic transitions with group differences in F2 slopes and locus equations; F2 slope correlated with intelligibility, but stimulation effects were heterogeneous.
Moreau et al^84^	Cross‐sectional	STN	11	19 [17–23]	69	NA	Low frequency (60 Hz) improved speech intelligibility, phonation, and pneumophonic coordination, although increased laryngeal resistance suggested possible compensatory vocal effort.
Morello et al^85^	Cross‐sectional	STN	19	14.6 ± 3.3	55.3 ± 9.8	NA	High frequency (130 Hz) improved vocal asthenia and instability but worsened articulation and dysarthria, highlighting divergent effects on voice and connected speech in PD.
Onder et al^68^	Cross‐sectional	STN	35	12.9 ± 5.5	59.8 ± 8.6	PD‐Med	STN‐DBS did not significantly affect VHI scores, whereas voice handicap correlated with disease duration and axial symptoms.
Phokaewvarangkul et al^50^	Cross‐sectional	STN	50	12.3 ± 4.6	60.8 ± 9.5	PD‐Med	STN‐DBS, especially high‐frequency stimulation and dorsal contact placement, reduced vocal intensity and increased stuttering; effects improved with lower frequencies.
Pina‐Fuentes et al^58^	Cross‐sectional	STN	13	16.0 ± 7.5	60.2 ± 11.1	NA	Adaptive DBS led to better speech intelligibility compared with conventional DBS. Benefits likely stem from reduced stimulation exposure with adaptive delivery.
Pinto et al^203^	Cross‐sectional	STN	26	14.8 ± 6.1	51.3 ± 7.2	NA	STN‐DBS improved articulatory forces, but speech intelligibility showed limited, transient benefit; long‐term decline suggests non‐dopaminergic degeneration contributes to dysarthria.
Pinto et al^73^	Cross‐sectional	STN	10	16.8 ± 3.3	53.7 ± 4.7	HCs	STN‐DBS improved phonatory and articulatory aspects, reversing abnormal brain activation patterns and restoring orofacial motor and cerebellar activity seen in parkinsonian dysarthria.
Putzer et al^204^	Cross‐sectional	STN	9	NA	61 ± 9	HCs	STN‐DBS variably affected articulation and phonation; group‐level data suggested worsened articulation, while individual effects differed.
Romann et al^76^	Cross‐sectional	STN	16	12.3 ± 4.0	57.3 ± 14.1	NA	STN‐DBS led to higher maximum fundamental frequency, indicating possible vocal instability, but had no significant effects on other acoustic voice measures.
Santens et al^52^	Cross‐sectional	STN	7	Mean 16.5 [9–30]	Mean 58.4 [39–68]	NA	Left‐sided STN stimulation significantly worsened prosody, articulation, and intelligibility, whereas right‐sided stimulation had no such effect; bilateral stimulation showed no overall speech difference compared with OFF stimulation.
Schulz et al^54^	Cross‐sectional	STN	12	15 ± 5	52 ± 11	NA	Bilateral or left‐sided STN‐DBS impaired speech, worsening VOT and other speech measures; right‐only or OFF‐stimulation conditions yielded better speech performance.
Sidtis et al^205^	Cross‐sectional	STN	7	11.3	61.2	PD‐Med HCs	STN‐DBS did not affect vowel or word durations in patients with PD, who showed preserved relational speech timing despite prolonged vowel durations.
Sidtis et al (2012)^206^	Cross‐sectional	STN	6	NA	58.2 [56–62]	PD‐Med	STN‐DBS mildly reduced speech intelligibility, especially for short, low‐context utterances, whereas repeated speech remained largely unaffected; external cues may aid speech performance.
Sidtis et al^207^	Cross‐sectional	STN	8	13.4 ± 3.3	57.5 ± 4.4	HCs	STN‐DBS reduced initial vowel space in sustained phonation; this effect correlated with worse speech ratings, although some voice parameters may improve because of enhanced glottal control.
Sidtis et al^109^	Cross‐sectional	STN	6	11.2	58 [56–62]	NA	DBS improved voice quality (higher F0 and HNR, lower shimmer) but reduced intelligibility and fluency, likely because of articulatory impairment and disrupted internal speech planning.
Sidtis et al^56^	Cross‐sectional	STN	7	NA	60.1 ± 7.2	NA	Both HFS and LFS reduced spontaneous speech intelligibility compared with DBS‐OFF, with greater decline under LFS; DBS increased voice and articulation abnormalities, with high variability.
Sidtis et al^111^	Cross‐sectional	STN	7	12 ± 3	63 ± 10	PD‐Med	STN‐DBS introduced conflicting activity patterns in left‐hemisphere speech‐related regions; higher stimulation amplitude on the left side was associated with slower speech rate.
Skodda et al^153^	Cross‐sectional	STN	14	12.1 ± 5.2	65.6 ± 6.8	PD‐Med	Patients with PD showed impaired syllable repetition stability regardless of treatment; STN‐DBS further worsened this instability, whereas l‐dopa had no effect.
Skodda et al^208^	Cross‐sectional	STN	24	16.7 ± 6.1	67.6 ± 7.1	HCs	STN‐DBS worsened syllable repetition stability, whereas l‐dopa partially improved pace consistency; effects likely reflect disrupted motor speech control and reduced automaticity.
Skodda et al^49^	Cross‐sectional	STN	38	12.2 ± 7.0	65.7 ± 7.9	HCs	STN‐DBS improved perceptual voice quality and showed trends toward better acoustic measures, but 21% of patients with baseline articulatory slurring and acceleration experienced worsened speech.
Svihlik et al^209^	Cross‐sectional	STN	23	17.8 ± 4.5	61.7 ± 5.0	HCs	STN DBS increased long‐term average spectrum spectral mean during reading, indicating improved speech under stimulation, whereas other measures and perceptual ratings showed no significant change.
Tanaka et al^75^	Cross‐sectional	STN	68	Male: 14.7 ± 3.9 Female: 15.5 ± 5.5	Male: 64.8 ± 8.3 Female: 67.2 ± 8.3	PD‐Med	STN‐DBS led to widespread voice impairments and poorer voice‐related QoL, especially in females; impairments improved OFF stimulation, suggesting direct adverse effects on vocal function.
Tanaka et al^210^	Cross‐sectional	STN	56	Male: 13.0 ± 5.1 Female: 13.4 ± 5.3	Male: 64.6 ± 9.2 Female: 68.5 ± 7.5	PD‐Med	STN‐DBS improved vowel articulation as reflected by increased vowel space area, but speech intelligibility and naturalness remained poorer than in medically treated patients.
Tanaka et al^211^	Cross‐sectional	STN	26	13.0 ± 4.1	65.9 ± 8.9	PD‐Med CA HCs	STN‐DBS increased speech rate instability, resembling cerebellar ataxia, but did not affect volume variability; turning stimulation OFF improved rate stability in most patients to levels seen in non‐DBS groups.
Törnqvist et al^53^	Cross‐sectional	STN	10	Median 13 [8–28]	Median 65 [60–75]	NA	Higher frequency and amplitude worsened intelligibility and articulation in some patients, whereas polarity changes had minimal impact.
Tripoliti et al^51^	Cross‐sectional	STN	14	15.6 ± 5	60 ± 6.5	NA	High‐voltage STN‐DBS significantly reduced speech intelligibility without affecting loudness; greater deterioration was linked to medially placed electrodes and current spread.
Tsuboi et al^13^	Cross‐sectional	STN	76	14.8 ± 5.3	66.1 ± 8.4	PD‐Med	STN‐DBS worsened overall speech intelligibility but improved voice tremor; strained voice and spastic dysarthria improved OFF stimulation, likely because of corticobulbar side effects from lateral electrode placement.
Tsuboi et al^47^	Cross‐sectional	STN	22	15.4 ± 3.9	65.2 ± 9.2	PD‐Med	STN‐DBS worsened laryngeal and voice function, increasing glottal abnormalities; incomplete closure correlated with breathiness, and false vocal fold hyperadduction with strain.
Valalik et al^79^	Cross‐sectional	STN	22	12.8 ± 4.5	59.5 ± 8.1	PD‐Med	STN‐DBS worsened phonation perceptually and acoustically, with nonlinear acoustic analysis detecting changes more sensitively than perturbation measures; overstimulation partially reversed these effects.
Van Lancker Sidtis et al^108^	Cross‐sectional	STN	7	11.9 [9–16]	58 [49–62]	NA	DBS improved voice quality, especially during repetition tasks, but had limited effect on fluency; dysfluencies decreased markedly with repetition and increased with longer disease duration.
Wertheimer et al^64^	Cross‐sectional	STN	287	16 ± 6.6	65 ± 9.0	PD‐Med	STN‐DBS significantly worsened speech from patients' perspectives, especially slurred and rapid speech, yet most still reported improved motor symptoms and overall QoL, highlighting a perceived trade‐off.
Xie et al^212^	Cross‐sectional	STN	11	[3–11]	58.2 ± 9.2	HCs	STN‐DBS had limited short‐term effects on speech overall. Female patients showed vowel‐specific acoustic changes, especially for /i/, suggesting gender‐related speech sensitivity.
Yilmaz et al^87^	Cross‐sectional	STN	16	NA	Mean 55 [38–70]	No control group	STN‐DBS at 130 Hz significantly improved articulation of vowel [a] in PD by increasing F1, suggesting better tongue and jaw movement; other frequencies showed nonsignificant trends.
Zhou et al^213^	Cross‐sectional	STN	19	9.3	63.8	PD‐Med	STN‐DBS significantly improved parkinsonian voice, as shown by reduced correlation dimension and improved perturbation measures; l‐dopa had limited effect, and no clear DBS– l‐dopa interaction was observed.
Gentil et al^214^	Cross‐sectional	STN/Vim	STN: 10 Vim: 4	STN: 11 ± 4 Vim: 13 ± 4	STN: 49 ± 9 Vim: 55 ± 7	HCs	STN‐DBS improved both motor and speech outcomes in PD, whereas Vim‐DBS improved motor function but worsened speech.
Jones et al^98^	Cross‐sectional	STN/GPi	STN: 7 GPi: 5	NA	61.3 ± 2.39	NA	DBS shortened speech reaction time during maintenance tasks, suggesting selective enhancement of speech motor program maintenance by left‐hemispheric stimulation.
Kim et al^166^	Cross‐sectional	STN/GPi	STN: 6 GPi: 3	[7.1–20.8]	72.1 ± 7.3	NA	DBS caused minimal overall changes in speech; some acoustic parameters changed significantly, with no significant differences in perceptual speech ratings between DBS‐ON and OFF.
Kopf et al^67^	Cross‐sectional	STN/GPi	STN: 12 GPi: 12	STN: 10.4 ± 4.0 GPi: 13.9 ± 6.8	STN: 63.7 ± 7.3 GPi: 65.8 ± 6.9	NA	VHI scores did not differ significantly between STN‐ and GPi‐DBS, although a trend favored STN; VHI correlated with intelligibility.
van Brenk et al^102^	Cross‐sectional	STN/GPi	STN: 10 GPi: 8	STN: mean 11.2 GPi: mean 13.4	STN: mean 64.6 GPi: mean 67.5	NA	Speech intelligibility and severity did not differ between STN‐ and GPI‐DBS or by stimulation status, despite small acoustic differences that lacked perceptual impact on functional communication.

*Note*: Studies are organized by DBS target, study design, and authors' names. For disease duration and age at assessment, values are reported as mean ± standard deviation or as median [range].

Abbreviations: PD, Parkinson's disease; DBS, deep brain stimulation; FU, follow‐up; STN, subthalamic nucleus; NA, not applicable; Med‐*on*, *on* medication; Med‐*off*, *off* medication; STIM‐ON, ON stimulation; ADL, activity of daily living; HC, healthy controls; SPL, sound pressure level; BMT, best medical therapy; VHI, Voice Handicap Index; QoL, quality of life; MPT, maximum phonation time; GRBAS, Grade, Roughness, Breathiness, Asthenia, Strain; LSVT LOUD, Lee Silverman Voice Treatment; GPi, internal globus pallidus; HNR, harmonics‐to‐noise ratio; MER, Microelectrode Recording; DDK, diadochokinesis; PPN, pedunculopontine nucleus; cZi, caudal zona incerta; HFS, high‐frequency stimulation; LFS, low‐frequency stimulation; Vim, ventral intermediate nucleus; UPDRS‐III, Unified Parkinson's Disease Rating Scale Part III; ET, essential tremor; AR, akinetic/rigid; TD, tremor dominant; CA, cerebellar ataxia.

**Table 2:** Studies reporting speech‐related outcomes for patients with ET

Authors	Study design	DBS target	No. of patients	Disease duration (yrs)	Age at assessment (yr)	Control group	Summary of key results
Erickson‐DiRenzo et al^130^	Prospective FU, 10 weeks	Vim	9	NA	NA	NA	DBS improved acoustic, physiological, and perceptual measures, reducing voice handicap.
Matsumoto et al^118^	Prospective FU, 51 days	Vim	18	NA	Mean 63.3 [55–84]	NA	Optimal speech outcomes post‐DBS were linked to precise ventral‐lateral posterior nucleus targeting; stimulation outside this target increased risk of voice tremor and dysarthria.
Obwegeser et al^128^	Prospective FU, 12 months	Vim	27	27 ± 15	73 ± 5.2	NA	Bilateral thalamic stimulation significantly reduced voice tremor but increased the risk of dysarthria, with more frequent programming adjustments required for speech issues in bilateral cases.
Pahwa et al^122^	Prospective FU, 1 year	Vim	9	NA	38.4 [20–58]	NA	Mild dysarthria was common post‐DBS; stimulation worsened speech in some, but effects were mostly reversible with parameter adjustment.
Patel et al^132^	Prospective FU, 7 months	Vim	10	NA	67 ± 6.7	NA	Bilateral Vim‐DBS significantly reduced vocal tremor and improved acoustic measures, indicating enhanced voice stability and quality.
Pulzke et al^124^	Prospective FU, 3 years	Vim	22	30.0 ± 14.3	70.3 ± 9 0.0	NA	Vim‐DBS improved voice tremor, especially with bilateral stimulation, but dysarthria occurred more frequently in bilateral cases; speech issues were generally mild and adjustable.
Taha et al^123^	Prospective FU, 10 months	Vim	ET: 15 PD: 2 MS: 2	NA	NA	NA	Bilateral Vim‐DBS significantly improved voice tremor in most patients, enabling conversation; dysarthria occurred in 30% but was often reversible with reprogramming, although sometimes at the cost of tremor control.
Becker et al^117^	Prospective FU, 7 months	Vim/PSA	13	24.2 ± 18.9	58.9 ± 17.0	HCs	Vim‐DBS prolonged syllable durations, indicating reduced articulation rate, although overall acoustic and VAS measures remained comparable with HCs.
Sun et al^215^	Prospective FU, 13 months	Vim/PSA	9	16.2 ± 11.4	56.0 ± 14.0	NA	PSA‐DBS significantly improved voice tremor and acoustic measures, whereas Vim‐DBS showed limited voice tremor benefit and more speech disturbances.
Low et al^216^	Prospective FU, 5 years	PSA	ET: 17 PD: 17	NA	NA	NA	Diffusion tensor imaging and tractography‐guided DBS improved tremor control and mobility with fewer speech issues.
Kundu et al^131^	Retrospective longitudinal FU, 1 year	Vim	20	NA	73 ± 7	NA	Vim‐DBS reduced voice tremor amplitude by 80%, with similar effects from unilateral and bilateral stimulation; anterior Vim targeting yielded better outcomes.
Mitchell et al^4^	Retrospective longitudinal FU, 1 year	Vim	Unilateral: 119 Bilateral: 39	Unilateral: 29.6 ± 17.5 Bilateral: 28.1 ± 16.0	Unilateral: 65.1 ± 9.3 Bilateral: 64.9 ± 9.5	NA	Unilateral Vim‐DBS improved voice tremor by 58.3%; bilateral DBS offered no additional voice benefit and increased dysarthria risk.
Ruckart et al^133^	Retrospective longitudinal FU, 6 months	Vim	5	NA	71 [47–81]	NA	DBS led to minimal voice changes, although vocal roughness increased; one voice tremor patient showed marked improvement across perceptual, acoustic, aerodynamic, and self‐reported voice measures.
Ruckart et al^134^	Retrospective longitudinal FU, 6 months	Vim	5	29 ± 26.2	72.8 ± 2.6	NA	Both unilateral and bilateral Vim‐DBS improved vocal tremor and voice‐related quality of life, with greater benefits in bilateral cases, while overall voice quality and dysarthria remained largely unchanged.
Sandstrom et al^16^	Retrospective longitudinal FU, 5 years	cZi	19	NA	NA	NA	Unilateral cZi‐DBS reduced voice tremor by 58% at 3 years and 67% at 5 years, with long‐term effectiveness varying by individual progression patterns in voice tremor.
Avecillas‐Chasin et al^217^	Cross‐sectional	Vim	7	13.2 ± 6.9	60.8 ± 8.1	NA	Unilateral dominant Vim‐DBS significantly reduced voice tremor compared with nondominant or no stimulation, highlighting lateralized effects in patients with bilateral implants.
Barbe et al^127^	Cross‐sectional	Vim	10	16.7 ± 11.7	69.3 ± 6.9	NA	Current‐shaping DBS reduced tremor (TRS, kinematics) but worsened subjective speech ratings.
Becker et al^17^	Cross‐sectional	Vim	16	17.3 ± 9.5	65.3 ± 11.0	NA	Bilateral Vim‐DBS caused more severe dysarthria than unilateral; severity correlated with electrode position and diadochokinetic rate, affecting intelligibility and patient‐rated speech outcomes.
Boogers et al^218^	Cross‐sectional	Vim	12	[15–60]	70.3 ± 10.5	NA	Symmetric biphasic pulses led to significantly higher DDK rates and showed trends toward better intelligibility and less dysarthria than cathodic pulses.
Carpenter et al^219^	Cross‐sectional	Vim	7	NA	[65–80]	NA	Vim‐DBS improved voice tremor in four of seven patients, although effects were unrelated to electrode site or stimulation parameters.
Cernera et al^18^	Cross‐sectional	Vim	8	[5–35]	[65–78]	NA	Continuous DBS reduced speech intelligibility and articulation rate, whereas responsive DBS preserved both.
Erickson‐DiRenzo et al^220^	Cross‐sectional	Vim	7	NA	NA	NA	Intraoperative voice assessment aided electrode placement; perceptual severity improved despite limited acoustic changes.
Kronenbuerger et al^135^	Cross‐sectional	Vim	12	35.5 ± 16.7	64.2 ± 10.6	ET‐Med HCs	DBS had no discernable effect on syllable repetition task.
Mucke et al^119^	Cross‐sectional	Vim/PSA	16	27.9 ± 17.6	64.7 ± 14.9	HCs	Although Vim‐DBS effectively treats tremor, it induces dysarthria‐like symptoms, reducing glottal abduction and articulation precision despite unchanged overall articulation rate.
Mucke et al^136^	Cross‐sectional	Vim/PSA	12	16.8 ± 12.4	61.6 ± 11.9	HCs	Thalamic DBS worsened preexisting speech coordination deficits, leading to slower articulation, increased frication, and glottal control impairments.
Petry‐Schmelzer et al^120^	Cross‐sectional	Vim/PSA	14	NA	65.4 ± 13	NA	Thalamic DBS induced dysarthria in 50% of patients with ET, with worsened intelligibility linked to stimulation of discriminative fibers near motor and cerebellar pathways.
Vogel et al^121^	Cross‐sectional	PSA	ET: 4 Dystonic tremor: 1 Cerebellar tremor: 1	[18–35]	[44–72]	NA	Optimal tremor settings often left speech unchanged, although subtle, patient‐specific speech timing and vocal control changes were detected with increased amplitude.
Hagglund et al^129^	Cross‐sectional	cZi	26	NA	68.1 ± 14.3	NA	cZi‐DBS reduced voice tremor in ~50% of patients with ET; bilateral stimulation was more effective than unilateral stimulation.
Sandstrom et al^125^	Cross‐sectional	cZi	11	NA	61.5 ± 6.6	NA	Left cZi‐DBS effectively reduced voice tremor in most patients with ET, matching or outperforming bilateral stimulation; excessive stimulation amplitude sometimes worsened tremor severity.
Sandstrom et al^221^	Cross‐sectional	cZi	37	33 ± 19	71 ± 10	NA	Unilateral cZi‐DBS reduced voice tremor in >70% of patients, with optimal effects from deep, posterior contacts; high‐amplitude stimulation could worsen or induce voice tremor.
Sandstrom et al^126^	Cross‐sectional	cZi	35	32 ± 18	70 ± 10	HCs	cZi‐DBS generally preserved speech intelligibility, although high‐amplitude unilateral stimulation impaired intelligibility in some, particularly older individuals or those with superior contact placements.
Sandstrom et al^222^	Cross‐sectional	cZi	14	32 ± 18	69 ± 11	NA	cZi‐DBS had no significant group‐level speech effects, but high‐amplitude stimulation impaired articulation; left‐sided stimulation was primarily responsible for speech deterioration.

*Note*: Studies are organized by DBS target, study design, and authors' names. For disease duration and age at assessment, values are reported as mean ± standard deviation or as median [range].

Abbreviations: DBS, deep brain stimulation; FU, follow‐up; Vim, ventral intermediate nucleus; NA, not applicable; PSA, posterior subthalamic area; ET, essential tremor; PD, Parkinson's disease; MS, multiple sclerosis; HC, healthy controls; VAS, visual analogue scale; cZi, caudal zona incerta; TRS, tremor rating scale; DDK, diadochokinesis.

**Table 3:** Studies reporting speech‐related outcomes for patients with dystonia

Authors	Study design	DBS target	Dystonia type	No. of patients	Disease duration (yrs)	Age at assessment (yr)	Control group	Summary of key results
Kupsch et al^140^	Prospective FU, 6 months	GPi	Generalized or segmental dystonia	Generalized: 24 Segmental: 16	Generalized: 16.3 ± 7.1 Segmental: 21.9 ± 7.9	Generalized: 33.8 ± 10.1 Segmental: 48.9 ± 13.5	NA	Bilateral pallidal stimulation improved movement, disability, and QoL in dystonia, although dysarthria was reported as an adverse event.
Sobstyl et al^141^	Prospective FU, 5 years	GPi	Meige syndrome	6	Mean 12.5 [9–22]	Mean 58.5 [48–67]	NA	GPi‐DBS improved speech/swallowing scores by 49% at 3 months and 39% at long‐term follow‐up, although the latter was not statistically significant, indicating possible waning benefit over time.
Volkmann et al^5^	Prospective FU, 1 year	GPi	Cervical dystonia	DBS: 32 Sham: 30	DBS: 14.9 ± 8.0 Sham: 14.8 ± 6.4	DBS: 57.1 ± 9.8 Sham: 56.6 ± 11.3	NA	Dysarthria, typically mild and slurred, occurred in seven patients and was the most common nonserious adverse event; it remained permanent in six of the affected cases.
Honey et al^223^	Prospective FU, 1 year	Vim	Spasmodic dysphonia	6	19.5 ± 11.3	65.2 ± 9.1	NA	DBS was safely performed in adductor SD patients, with trends toward improved voice and QoL.
Ouyang et al^224^	Prospective FU, 1 year	STN	Meige syndrome	15	7.2 ± 4.4	60.2 ± 6.9	NA	STN‐DBS improved speech and swallowing scores by 59.1%, indicating significant benefit for these motor symptoms compared with baseline.
Danielsson et al^146^	Retrospective longitudinal FU, 46 months	GPi	DYT‐THAP1	14	Median 9 [2–19]	Median 18 [8–57]	NA	Pallidal DBS reduced dystonia severity by 58% in DYT‐THAP1 patients, mainly improving trunk and limb symptoms, with limited effects on speech and swallowing.
Finger et al^225^	Retrospective longitudinal FU, 1 year	GPi	DYT1: 4 Generalized dystonia: 4 Cervical dystonia: 6	14	23.2 ± 12.1	62.6 ± 16.3	NA	GPi‐DBS improved speech intelligibility and voice quality in primary dystonia over 12 months, although responses varied.
Groen et al^147^	Retrospective	GPi	DYT‐THAP1: 5	5	NA	37.8 ± 23.4	NA	GPi‐DBS improved motor function in early‐onset DYT6 dystonia, although speech benefits were limited.
Hao et al^21^	Retrospective longitudinal FU, 1 year	GPi	Meige syndrome	22	5.5 ± 4.1	57.9 ± 6.8	NA	GPi‐DBS improved motor symptoms, speech, and QoL, with 78% motor and 61% speech‐related improvement at 12 months.
Isaias et al^137^	Retrospective longitudinal FU, 8 years	GPi	Primary generalized	30	Mean 14	28 ± 17	NA	Speech and swallowing dystonia showed limited DBS responsiveness, with some patients experiencing worsening symptoms.
Limotai et al^145^	Retrospective longitudinal FU, 1 year	GPi	Meige syndrome	6	11.3 ± 5.9	67.7 ± 14.6	NA	GPi‐DBS improved dystonia severity in craniocervical regions, but speech and swallowing difficulties persisted.
Reese et al^138^	Retrospective longitudinal FU, 38 months	GPi	Meige syndrome	12	8.6 ± 4.4	64.5 ± 4.4	NA	GPi‐DBS improved speech/swallowing subscores, with greater benefit at long‐term follow‐up; dysarthria occasionally occurred at supratherapeutic stimulation intensities.
Ren et al^142^	Retrospective longitudinal FU, 36 months	GPi	Meige syndrome	13	9.2 ± 2.1	46.9 ± 7.2	NA	GPi‐DBS significantly improved speech/swallowing scores; dysarthria occurred transiently in one case and resolved with parameter adjustment.
Sensi et al^139^	Retrospective longitudinal FU, 58 months	GPi	Primary segmental dystonia	11	18.5 [12–25]	43.7 [23–63]	NA	GPi‐DBS led to marked long‐term improvement in speech and swallowing, with speech intelligibility improving by ~60% at 3 years, especially in previously nearly anarthric patients.
Tian et al^143^	Retrospective longitudinal FU, 15 months	GPi	Meige syndrome	40	3.8 ± 2	57.3 ± 7.3	NA	Speech and swallowing function significantly improved after GPi‐DBS, reaching a 90% improvement at 2 years compared with baseline.
Tian et al^144^	Retrospective longitudinal FU, 30 months	STN; GPi	Meige syndrome	STN: 11 GPi: 6	4.7 ± 3.2	56 ± 8.4	NA	Both STN‐ and GPi‐DBS improved speech and swallowing function in primary Meige syndrome, with comparable beneficial effects on BFMDRS subscores.
Evidente et al^226^	Retrospective	Vim	Spasmodic dysphonia	3	12.3	70	NA	Bilateral Vim‐DBS improved adductor SD and voice tremor; SD required higher stimulation parameters.
Pauls et al^149^	Cross‐sectional	GPi	Generalized dystonia: 3 Cervical dystonia: 3 Segmental dystonia: 4	10	17.7 ± 14.5	53.3 ± 16.1	NA	GPi‐DBS increased speech pauses and slowed syllable repetition with posterior contacts, indicating stimulation‐location effects on speech motor control in dystonia.
Risch et al^20^	Cross‐sectional	GPi	Primary dystonia	15	15.5 ± 11.3	60.7 ± 10.1	HCs	Articulation rate slightly improved with stimulation, but individual cases showed variable outcomes, including both improvement and stimulation‐induced dysfluency.
Rusz et al^11^	Cross‐sectional	GPi	Generalized: 12 Cervical: 7	19	13.5 ± 6.2	49.2 ± 17.8	HCs	GPi‐DBS improved hyperkinetic dysarthria in 47% of patients with dystonia but worsened hypokinetic dysarthria and fluency in others, with adverse effects linked to high intensity and medial contact placement.
Rusz et al^148^	Cross‐sectional	GPi	Generalized: 10 Cervical: 8	18	13.6 ± 6.4	48.6 ± 18.1	HCs	GPi‐DBS did not significantly affect articulation or DDK rate; speech remained slower than HCs regardless of stimulation, with no association to electrode location or intensity.

*Note*: Studies are organized by DBS target, study design, and authors' names. For disease duration and age at assessment, values are reported as mean ± standard deviation or as median [range].

Abbreviations: DBS, deep brain stimulation; FU, follow‐up; NA, not applicable; QoL, quality of life; GPi, internal globus pallidus; Vim, ventral intermediate nucleus; SD, spasmodic dysphonia; STN, subthalamic nucleus; BFMDRS, Burke–Fahn–Marsden dystonia rating scale; HC, healthy controls; DDK, diadochokinesis.

### Methodological Landscape of Speech

Common perceptual end points were non‐speech‐specific scales (eg, UPDRS‐III item 18 or Burke–Fahn–Marsden dystonia rating scale [BFMDRS] speech/swallowing subscore), speech intelligibility, and voice quality scales (eg, Grade, Roughness, Breathiness, Asthenia, Strain [GRBAS]).

**FIG. 1:**
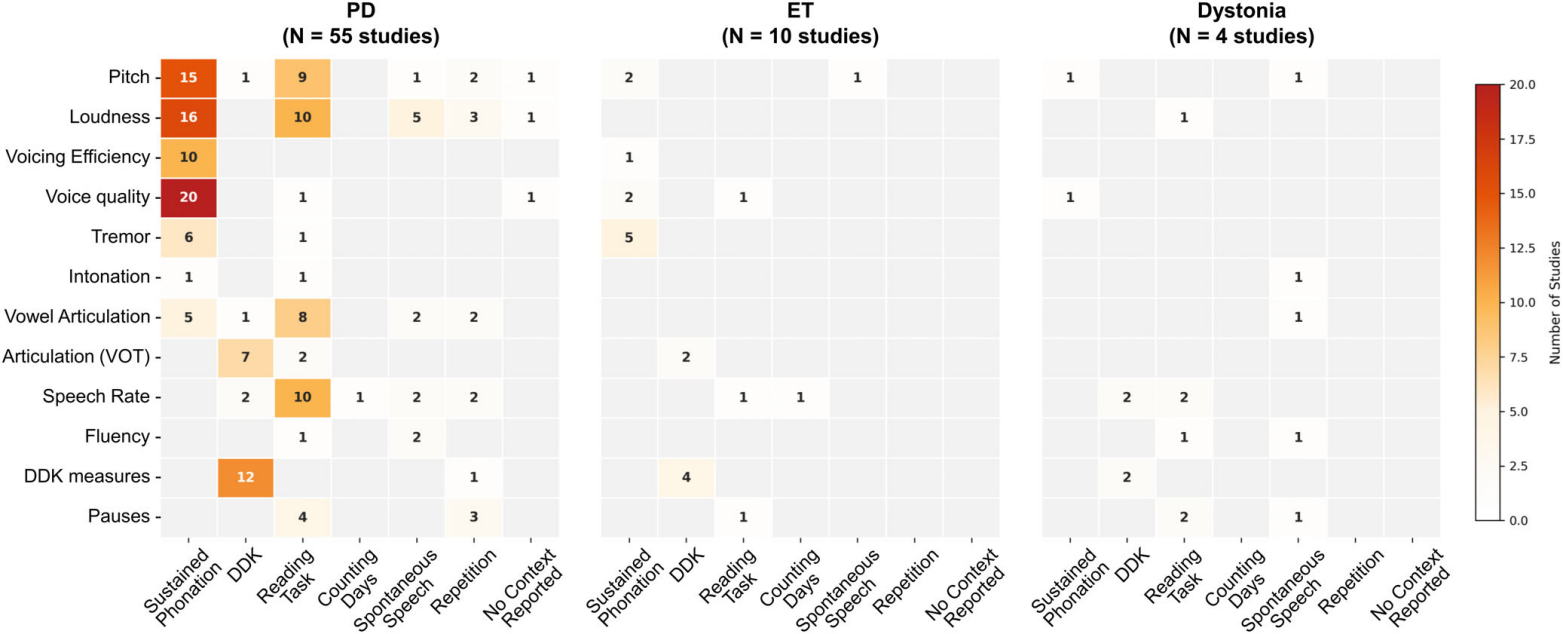
Acoustic measures and speech‐elicitation contexts across included studies. Heatmap showing the number of studies reporting each acoustic measure domain stratified by speech‐elicitation context and disorder. Panels are arranged by sample size: PD (n = 55), ET (n = 10), and dystonia (n = 4). Sixty‐three distinct acoustic variables were identified and grouped into 12 measurement domains derived from seven task contexts: sustained phonation, DDK, reading task of sentence/paragraph, counting days of the week or months, spontaneous speech, repetition of word/phrase/sentence after an audio cue, and studies with no context reported. Color intensity reflects the absolute number of studies; empty cells denote combinations not assessed in any included study. For detailed definitions of acoustic measures, see Table S2. DDK, diadochokinesis; ET, essential tremor; PD, Parkinson's disease; VOT, voice‐onset time.

We identified 63 distinct acoustic measures across 12 acoustic dimensions derived from six task settings (phonation, reading, diadochokinesis [DDK], spontaneous speech, repetition of words or phrases, and automatic speech; Fig. 1; Table S2). Sustained phonation was the most commonly used task, whereas connected or spontaneous speech was the least frequent (Fig. 1). Recording setups (microphone type/position, sampling rates), software, and preprocessing varied widely, and longitudinal acoustic datasets were limited: none in dystonia. This methodological spread limited cross‐study comparability.

PROs were mostly dominated by the Voice Handicap Index (VHI),^33^ with occasional use of the DBS Impairment Scale^34^ or bespoke telephone questionnaires.

#### Parkinson's Disease

##### Meta‐analysis of STN‐DBS Versus Best Medical Treatment (Speech Intelligibility)

Four prospective cohorts from Germany, France, the UK, and Japan were included (252 patients with DBS and 125 medically treated PD controls; per study, n = 43–145).^2^, ^3^, ^6^ Each cohort reported speech intelligibility scores presurgery and at 1 to 2 years of follow‐up in the DBS‐ON/Med‐*on* states (Table S3).

**FIG. 2:**
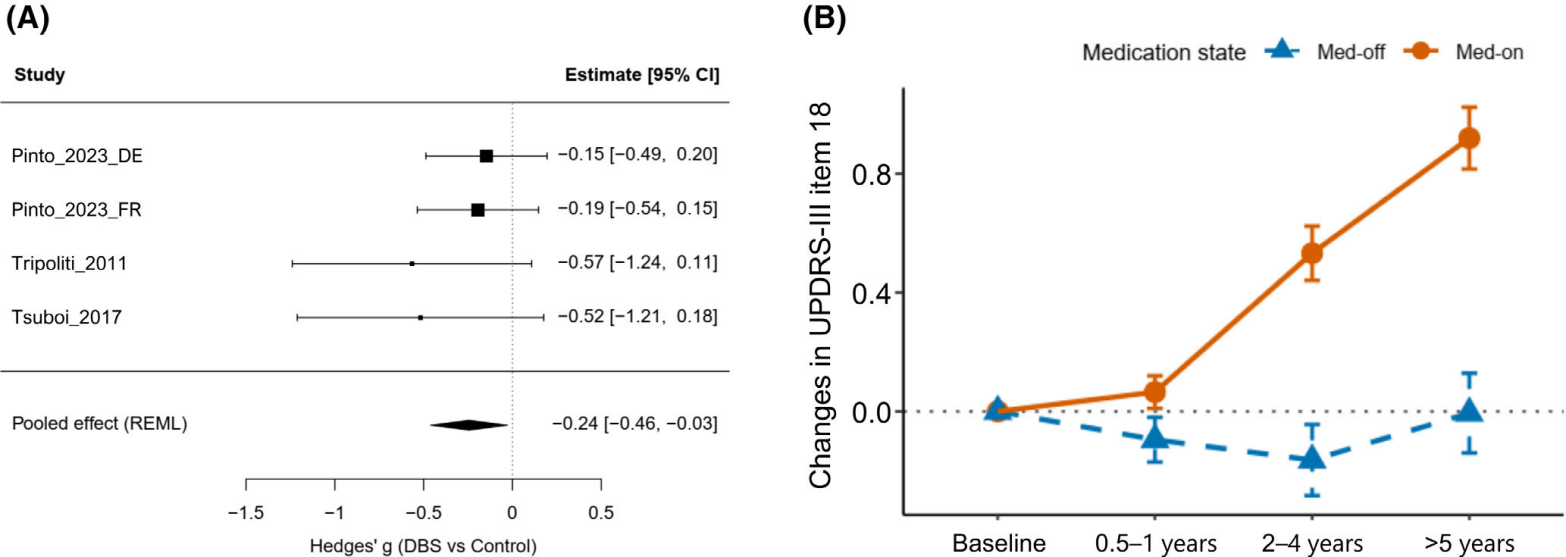
Meta‐analysis of speech outcomes after subthalamic nucleus deep brain stimulation (STN‐DBS) in Parkinson's disease. (**A**) Forest plot showing study‐level and pooled estimates comparing speech intelligibility between STN‐DBS and medically treated groups. (**B**) Longitudinal changes in Unified Parkinson's Disease Rating Scale Part III (UPDRS‐III) item 18 (speech) in STN‐DBS cohorts. Changes from baseline (positive = worse) are shown separately for the on medication (Med‐*on*; *orange circles*) and off medication (Med‐*off*; *blue triangles*) states. Error bars indicate 95% confidence intervals (CIs). REML, restricted maximum likelihood. [Color figure can be viewed at wileyonlinelibrary.com]

Pooled across studies, STN‐DBS was associated with significantly worse intelligibility versus medical therapy (Hedges' *g* = −0.24, 95% confidence interval: −0.46 to −0.03, *P* = 0.027; Fig. 2A; Table S4). Between‐study heterogeneity was negligible (*Q* = 1.87, *P* = 0.60, *I* ^2^ = 0%), and fixed‐ and random‐effects models yielded virtually identical estimates (Table S5). The effect size was small but consistently negative, indicating a modest but unfavorable impact of STN‐DBS on speech intelligibility.

Robustness checks supported these findings: leave‐one‐out analyses preserved the direction and magnitude of the effect (pooled *g* range: −0.21 to −0.31; Table S6), and varying the pre–post correlation from 0.3 to 0.7 produced stable estimates (Table S7). We detected no evidence of small‐study effects or publication bias (symmetric funnel plot; Egger's test *z* = −1.35, *P* = 0.18; Fig. S2).

##### Meta‐analysis of Longitudinal Speech Changes in PD (UPDRS‐III Item 18)

Longitudinal changes in UPDRS‐III item 18 (Δ from baseline; positive = worse) were synthesized from 12 STN‐DBS studies (12 DBS‐ON/Med‐*on*, 10 DBS‐ON/Med‐*off*; 43 effect sizes) (Tables S8 and S9).^35^, ^36^, ^37^, ^38^, ^39^, ^40^, ^41^, ^42^, ^43^, ^44^, ^45^, ^46^ In the Med‐*off* state, meta‐regression showed no significant longitudinal change over 5 years (slope = +0.002 per month, *P* = 0.50), whereas the Med‐*on* slope was significantly positive (slope = +0.016 per month, *P* < 0.001); the time‐by‐state interaction was significant (Δ = +0.014 per month, *P* = 0.0013), indicating a steeper decline in Med‐*on* (Fig. 2B).

Consistent with these slopes, pooled Δ values (Δ from baseline) increased in Med‐*on* across follow‐up: +0.065 at 0.5 to 1 year, +0.532 at 2 to 4 years, and +0.920 beyond 5 years. By contrast, Med‐*off* remained near or slightly better than baseline: −0.095 at 0.5 to 1 year, −0.164 at 2 to 4 years, and −0.006 beyond 5 years (Table S10).

Funnel plot inspection did not suggest substantial asymmetry (Fig. S3). Egger's regression test at the effect‐size level was nonsignificant (*P* = 0.28); when effects were aggregated at the study level, the test remained nonsignificant but approached the threshold (*P* = 0.054; Table S11).

##### Perceptual Speech Outcomes

Across the literature, STN‐DBS was associated with worse perceptual speech performance than medication‐only management, consistent with our meta‐analysis.^2^, ^3^, ^6^, ^13^, ^40^, ^47^, ^48^, ^49^, ^50^ In addition, cross‐sectional and acute ON↔OFF/crossover studies repeatedly showed worse speech intelligibility and UPDRS III item 18 with stimulation ON.^2^, ^3^, ^13^, ^40^, ^48^, ^49^, ^50^, ^51^, ^52^, ^53^, ^54^, ^55^, ^56^, ^57^, ^58^ Longitudinal STN cohorts further demonstrated progressive worsening of UPDRS‐III item 18 over time, with a greater decline during Med‐*on* than Med‐*off*.^2^, ^3^, ^6^, ^35^, ^36^, ^37^, ^39^, ^43^, ^45^, ^46^, ^59^, ^60^ Taken together, these findings suggest that longitudinal worsening of perceptual speech function reflects both stimulation‐related effects and progressive disease‐related decline, becoming more apparent with longer postoperative duration.

##### Patient‐Reported Outcomes

Thirteen studies investigated PROs of speech after DBS, most commonly using the VHI.^9^, ^13^, ^47^, ^61^, ^62^, ^63^, ^64^, ^65^, ^66^, ^67^, ^68^, ^69^, ^70^ A few employed a telephone‐based questionnaire^62^ or the DBS Impairment Scale.^63^ Across studies, patients consistently reported substantial negative impact of speech impairment on social interaction, daily functioning, and work participation. One qualitative interview study with four patients further highlighted communication barriers related to impaired auditory feedback, mental fatigue, and perceived inevitability of limb motor deterioration.^71^

##### Acoustic Outcomes for Speech

Across the 55 studies that applied acoustic analysis (Table 1, Fig. S4A), outcomes were heterogeneous but showed consistent trends. Most cohorts targeted the STN (~85%), while GPi, PSA/cZi, and PPN data were sparse. Short‐term improvements in phonatory parameters, such as increased loudness and pitch stability, were observed mainly during sustained phonation or reading tasks.^2^, ^8^, ^72^, ^73^, ^74^, ^75^, ^76^ In contrast, connected speech tasks frequently demonstrated deterioration in articulation, prosody, and fluency, including delayed voice‐onset time (VOT), reduced vowel space, unstable formants, and stuttering‐like dysfluency.^2^, ^43^, ^49^, ^50^, ^66^, ^77^

Longitudinal data showed that early phonatory gains rarely persisted beyond 1 to 3 years, whereas articulation and intelligibility declined progressively.^10^, ^43^, ^66^, ^76^, ^77^, ^78^, ^79^ These effects tended to be stronger in the Med‐*on* states, paralleling perceptual findings. Methodological variability in recording and metric selection further limited reproducibility. Collectively, the data support a dissociation between transient phonatory improvements and decline in articulation, prosody, and fluency.

##### Nonstandard Stimulation Settings

Seventeen studies examined parameter effects on speech. Across short‐term trials, low frequencies (~60 Hz) were often associated with transient benefits for speech (and gait),^9^, ^50^, ^56^, ^63^, ^72^, ^80^, ^81^, ^82^, ^83^, ^84^, ^85^, ^86^, ^87^, ^88^ whereas higher frequencies tended to reduce intelligibility or produce strained voice quality. However, longitudinal studies indicated that the acute positive effect on speech and gait/balance of low frequency was followed by subsequent worsening of tremor requiring a return to high‐frequency stimulation.^9^

Short pulse width (~30 μs) may widen the therapeutic window in STN‐DBS,^89^, ^90^, ^91^, ^92^ but had a minimal impact on speech.^89^, ^90^, ^91^, ^92^ A well‐designed study demonstrated reduced dysfluency without clear gains in overall intelligibility.^92^ Speech impairments were strongly amplitude dependent, often emerging at lower amplitudes than those needed for optimal motor control.^51^, ^93^, ^94^ This highlights a clinical trade‐off between motor benefit and speech preservation.

Notably, two adaptive DBS studies demonstrated that speech intelligibility was better preserved with stimulation dynamically modulated by beta‐band activity than with conventional continuous stimulation.^58^, ^95^ Together, speech outcomes depend sensitively on frequency, pulse width, and amplitude, with adaptive or individualized programming showing potential to mitigate dysarthria.

##### Targets Other than the STN

In contrast with the extensive STN literature, evidence for GPi is sparser,^39^, ^67^, ^88^, ^96^, ^97^, ^98^, ^99^, ^100^, ^101^, ^102^, ^103^ without consistent perceptual advantage over STN. Quantitative multidimensional assessment indicated that GPi stimulation caused deterioration in articulation clarity, voice quality, and prosody.^96^ Small head‐to‐head cohorts comparing STN and GPi found no between‐target differences in intelligibility or overall perceptual severity under ON↔OFF testing,^102^ and cross‐sectional PROs likewise showed no target effect.^67^, ^68^ Longitudinal GPi datasets remain limited,^96^, ^101^, ^103^ precluding firm conclusions. Overall, current evidence does not support GPi DBS as reliably superior to STN for speech outcomes.

For cZi/PSA, multiple blinded listener studies reported worse intelligibility and articulation with ON stimulation versus OFF,^104^, ^105^ and an acoustic study noted reduced pitch variability and prosodic alterations.^106^ Compared with typical STN series, these data suggest a less favorable speech profile for cZi/PSA, although sample sizes are modest.

Regarding thalamic Vim,^97^ long‐term multicenter surveillance identified speech disturbance among common stimulation‐related adverse effects,^103^ but formal perceptual outcomes remain scarce. PPN cohorts are small and heterogeneous,^107^ with variable changes in intelligibility and prosody and no consistent target‐level signal. Further target‐specific, longitudinal, and head‐to‐head trials are needed.

##### Phenomenology and Pathophysiology of DBS‐Related Speech

DBS‐related dysarthria commonly presented as mixed dysarthria combining hypokinetic, spastic, or ataxic components.^2^, ^13^, ^14^, ^22^, ^59^ Furthermore, stuttering‐like disfluency was observed to arise de novo, with partial reversibility after stimulation reduction or cessation.^3^, ^13^, ^50^, ^62^, ^108^, ^109^ In parallel, hypokinetic‐sounding speech or speech akinesia was reported after both STN and GPi stimulation.^13^, ^57^, ^96^ Longitudinally, dysarthria commonly emerged or progressed over 1 to 3 years postoperatively,^10^, ^43^, ^77^ suggesting contributions from maladaptive neuroplasticity or progressive network reorganization beyond immediate stimulation effects. Among clinical factors, longer disease duration rather than age or baseline motor severity has been linked to greater vulnerability.^7^, ^13^, ^47^

Tractography‐informed mapping supported a dual‐network mechanism. In STN‐DBS for PD, stimulation spread to lateral corticobulbar and posteromedial cerebellothalamic pathways independently increased dysarthria risk.^70^ Involvement of corticobulbar fibers likely contributed to spastic qualities,^2^, ^7^, ^13^, ^15^, ^73^, ^110^, ^111^ whereas cerebellothalamic fiber involvement aligned with prosodic disturbances and dysfluency.^13^, ^48^, ^66^, ^73^, ^76^, ^77^ The spatial relationships of these pathways varied across other DBS targets, motivating pathway‐aware programming strategies.

Functional imaging indicated opposing patterns of modulation: improvement coincided with normalization of motor‐cerebellar underactivation, whereas worsening aligned with exaggerated and disorganized cortical activation.^73^, ^111^ Clinically, left‐sided stimulation more often impaired prosody and articulation than right‐sided stimulation, consistent with the lateralization of speech‐language networks.^2^, ^7^, ^44^, ^52^, ^57^, ^112^, ^113^

Taken together, DBS‐related dysarthria likely arises from the interplay of tract‐mediated effects, network‐level alterations, hemispheric lateralization, and longer‐term maladaptive plasticity, producing distinct but often overlapping phenotypes whose recognition may guide programming strategies.

##### Speech Therapy After DBS

Two studies evaluated behavioral therapy (Lee Silverman voice treatment [LSVT LOUD]) in post‐DBS patients.^65^, ^114^ Both reported limited benefit, in contrast with the established efficacy of such interventions in medically managed PD patients.^115^ This suggests that DBS‐related speech disorders may reflect a different pathophysiology that is less responsive to compensatory motor learning.^116^

#### Essential Tremor

##### Overview of Included Studies

Of the 32 ET studies (Table 2), 24 investigated Vim stimulation, with fewer addressing the cZi/PSA. Five studies examined exclusively bilateral stimulation,^117^, ^118^, ^119^, ^120^, ^121^ and several directly compared unilateral versus bilateral approaches.^4^, ^17^, ^104^, ^122^, ^123^, ^124^, ^125^, ^126^ Follow‐up durations ranged from 2 months to 5 years.

##### Perceptual Speech Outcomes

Across ET studies, a consistent dissociation was observed between robust voice tremor suppression and the risk of stimulation‐induced dysarthria.^16^, ^17^, ^118^, ^120^, ^121^, ^125^, ^126^, ^127^ Unilateral Vim DBS commonly improved voice tremor while maintaining overall clarity. In contrast, bilateral Vim stimulation provided little additional benefit but was strongly associated with dysarthria, manifesting as slurred or slowed speech, prolonged syllable duration, and reduced intelligibility.^4^, ^17^, ^118^, ^119^, ^122^, ^123^, ^124^, ^128^ The resulting pattern often resembled ataxic or mixed spastic‐ataxic dysarthria.^118^, ^126^, ^127^

PSA/cZi stimulation also reduced voice tremor and generally preserved intelligibility, with benefits durable up to 5 years.^16^, ^125^, ^126^, ^129^ Nevertheless, adverse articulatory effects still occurred at high‐amplitude settings or with more superior contact locations.^16^, ^118^, ^126^

##### Acoustic Outcomes

Across the 10 studies reporting acoustic analysis (Fig. S4B), a dissociation between phonatory and articulatory effects of DBS was observed. Voice‐tremor indices (eg, amplitude tremor intensity index or frequency tremor intensity index) and measures of phonatory stability generally improved after Vim or PSA/cZi stimulation, with some benefits sustained long term.^16^, ^125^, ^129^, ^130^, ^131^, ^132^, ^133^, ^134^ In contrast, temporal and articulatory parameters (such as syllable duration, diadochokinetic rate, and formant precision) often worsened, particularly under bilateral Vim stimulation or high‐amplitude settings.^17^, ^118^, ^119^, ^127^, ^135^, ^136^ Collectively, these findings indicate that DBS stabilizes voice tremor while compromising fine articulatory control.

#### Dystonia

##### Overview of Included Studies

Of the 21 dystonia studies (Table 3), most targeted the GPi, with smaller numbers investigating the STN or Vim. Cohorts were heterogeneous, including generalized dystonia, cervical dystonia, Meige syndrome, and genetic forms of dystonia, complicating direct comparison across series. Follow‐up durations ranged from 6 months to more than 5 years.

##### Perceptual Speech Outcomes

Perceptual outcomes were most often evaluated with the BFMDRS speech/swallowing subscore, whereas speech‐specific measures were rare. Approximately half of longitudinal GPi cohorts reported improvement,^137^, ^138^, ^139^, ^140^, ^141^, ^142^, ^143^, ^144^ but outcomes were highly variable both across and within studies.^20^, ^145^, ^146^, ^147^, ^148^ In Meige syndrome, benefit was often sustained over years,^21^, ^138^, ^141^, ^142^, ^143^, ^144^ whereas genetic forms such as DYT‐THAP1 and DYT‐KMT2B showed little or no speech response.^11^, ^146^, ^147^

Several studies highlighted stimulation‐related determinants: higher intensity was associated with hypokinetic‐like dysarthria,^11^, ^149^ and posterior or medial GPi contacts predicted worsening of articulation.^148^, ^149^ Taken together, speech outcomes appear to depend not only on phenotype but also on electrode placement and programming strategy.

##### Acoustic Outcomes

Only four cross‐sectional studies examined acoustic aspects (Fig. S4C).^11^, ^20^, ^145^, ^148^ These examined articulation rate, DDK, fluency, and acoustic markers spanning hyperkinetic and hypokinetic dysarthria. Findings were heterogeneous: some reported no ON‐OFF differences,^20^, ^148^ whereas others showed improvements in irregular pitch and strained voice quality alongside worsening of speech rate or fluency at higher stimulation intensity or at less favorable electrode locations.^11^, ^149^ Notably, one study highlighted a dualistic effect, with some patients improving while others deteriorated under identical stimulation conditions.^11^ Overall, acoustic analyses showed subtle stimulation‐related changes not consistently captured by clinical rating scales, but the small number of studies and variable methodologies limit firm conclusions.

#### Active Contact Localization

**FIG. 3:**
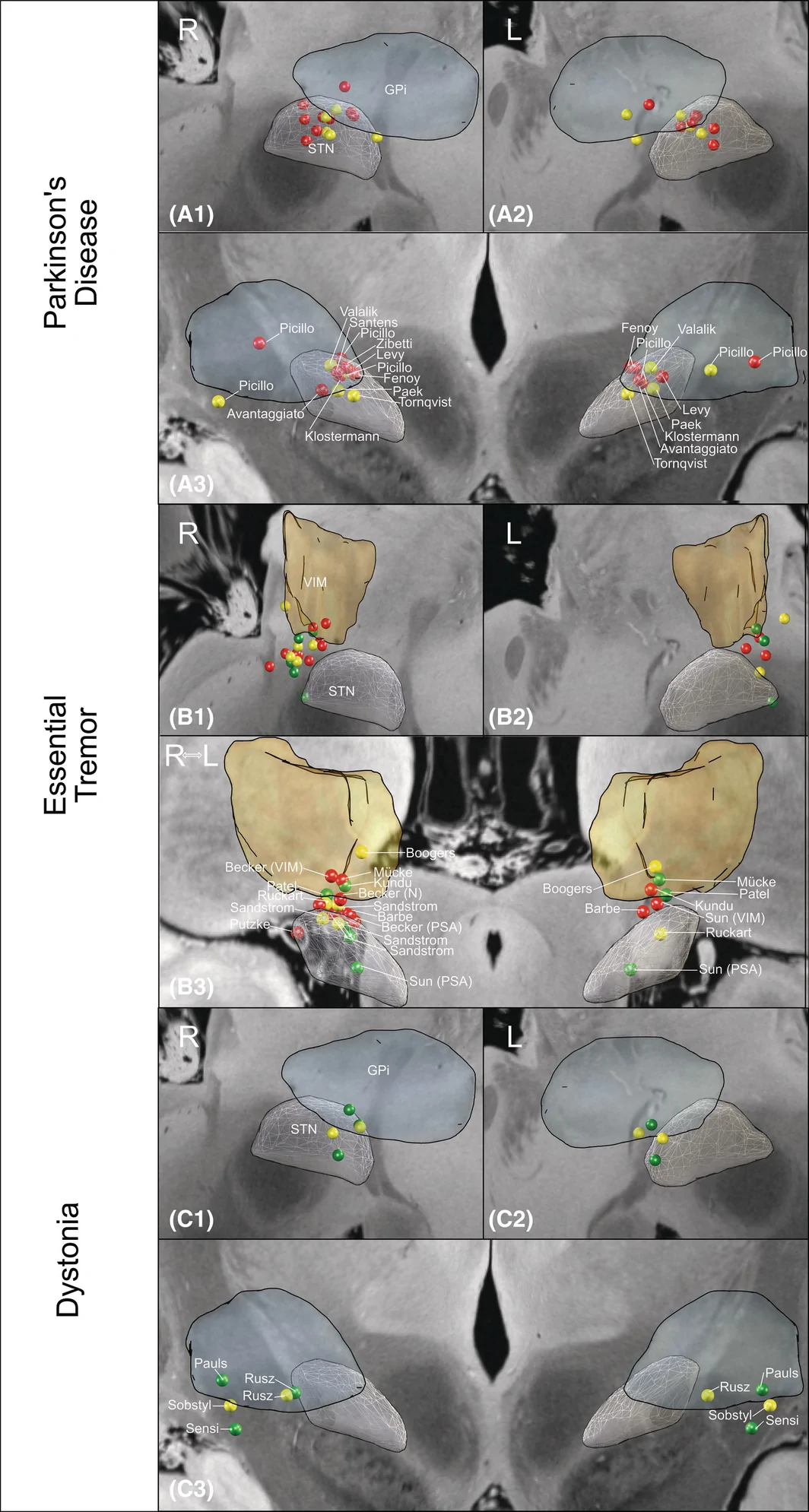
Active contact localization. This figure shows reported active‐contact locations for studies in Parkinson's disease (A1–A3), essential tremor (B1–B3), and dystonia (C1–C3). For each disorder, lateral views (A1–A2, B1–B2, C1–C2) and a coronal view (A3, B3, C3) are provided. Contacts are displayed relative to major anatomical structures: STN (fishnet), GPi (blue), and thalamus/Vim (tan). Each marker represents a study‐level or averaged contact position as reported in the corresponding publication. Color indicates the stimulation effect on speech (green = improvement; yellow = no clear change; red = worsening). Because several studies mirrored coordinates to the right hemisphere or provided only mean values, right‐sided markers appear proportionally larger. Across disorders, localization data were heterogeneous in format and precision, limiting direct cross‐study comparison; thus, the figure provides a qualitative overview rather than a quantitative spatial analysis. GPi, globus pallidus internus; PSA, posterior subthalamic area; STN, subthalamic nucleus; Vim, ventral intermediate nucleus.

Across all 184 included studies, 57 provided some information on electrode localization (23/131 of PD, 12/32 of ET, and 13/21 of dystonia studies). Reporting was frequently averaged or indirect, and only a minority provided precise stereotactic coordinates in AC–PC or MNI space. Figure 3A–C summarizes available localizations for PD, ET, and dystonia, respectively, in lateral and coronal projections relative to the major anatomical structures targeted (STN, GPi, and thalamus/Vim). Each marker represents average contact positions and their reported effects on speech. Because several studies mirrored contact coordinates to the right hemisphere or reported only mean positions, right‐sided markers appear disproportionately concentrated. These inconsistencies limit the feasibility of any quantitative spatial analysis; accordingly, Figure 3 should be interpreted as a qualitative overview.

## Discussion

This systematic review and meta‐analysis of 184 studies provided the first comprehensive cross‐disease synthesis of speech outcomes after DBS in PD, ET, and dystonia. Although DBS effectively controls motor symptoms across these disorders, it frequently compromises speech function. In PD, our meta‐analysis demonstrated that routine STN‐DBS is associated with poorer speech intelligibility compared with best medical treatment, with a steeper longitudinal decline in the Med‐*on* than Med‐*off* state. In ET, Vim or PSA/cZi stimulation consistently suppressed voice tremor but compromised articulatory precision, particularly with bilateral stimulation. Speech outcomes after GPi‐DBS in dystonia were highly variable, likely reflecting phenotypic and genetic heterogeneity.

Across disorders, a consistent dissociation emerged between sustained phonation and connected speech performance. Improvements in sustained phonation acoustics contrasted with declines in connected speech, including articulatory precision and prosodic regulation. This dissociation highlights the selective vulnerability of complex, integrative speech motor tasks and supports prioritizing connected speech and intelligibility measures in post‐DBS assessment. Together, these findings underscore a disease‐ and target‐specific trade‐off between motor benefit and speech modulation, emphasizing the need to systematically integrate speech metrics into DBS programming and follow‐up. Accordingly, we propose a practical, speech‐conscious programming framework and key directions for future research.

### Underlying Mechanisms of Speech Modulation by DBS

DBS exerts divergent effects on speech.^7^, ^11^, ^13^ In many patients, stimulation improves phonatory stability, loudness, and tremor‐related voice fluctuations, particularly during sustained phonation or brief speech production. These benefits likely reflect partial normalization of pathologically altered basal ganglia–cerebellar network activity, resulting in more stable laryngeal motor output.^138^, ^139^ However, DBS may impair connected speech intelligibility and prosody, resulting in dysarthric or disfluent speech patterns. Converging evidence suggests that these adverse effects arise from interacting mechanisms, including: (1) current spread to specific fiber pathways, (2) altered network connectivity within target nuclei, and (3) maladaptive neuroplasticity superimposed on disease‐specific pathophysiology.^2^, ^10^, ^13^, ^15^, ^43^, ^70^, ^111^ Mixed presentations are common, with tract‐mediated and connectivity‐driven features often cooccurring on a background of preexisting disease‐related dysarthria.^3^, ^13^, ^15^ Accordingly, distinct phenotypic signatures and their underlying pathophysiology should guide speech‐conscious programming strategies.

Contact topology studies have identified two recurrent tract‐mediated phenotypes. In STN‐DBS for PD, lateral current spread into corticobulbar fibers and posteromedial spread into cerebellothalamic pathways have been associated with spastic‐type and ataxic phenotypes.^2^, ^7^, ^13^, ^15^, ^38^, ^48^, ^66^, ^73^, ^76^, ^77^, ^111^ Supporting this localization, pyramidal tract side effects of STN stimulation include dysarthria and tetanic contractions of the face and upper limb.^150^, ^151^, ^152^ These signs are consistent with high‐frequency overactivation of corticobulbar or corticospinal fibers. In this context, we use the term spastic‐type rather than spastic dysarthria to avoid implying a lesion‐based pathophysiology. In parallel, prolonged and more variable VOT has been reported after both Vim‐DBS in ET and STN‐DBS in PD, particularly with medially positioned contacts suggesting involvement of cerebellothalamic fibers.^54^, ^81^, ^106^, ^109^, ^112^, ^119^, ^136^, ^153^ These tract‐related phenotypes often respond predictably to geometry‐based programming strategies, such as shifting active contacts away from the implicated fibers or applying directional current steering.^154^, ^155^, ^156^, ^157^

By contrast, hypokinetic speech features and stuttering‐like disfluencies are less consistent with isolated fiber capture and more suggestive of altered network dynamics within target nuclei.^13^, ^15^, ^50^ Hypokinetic‐sounding speech has been reported after STN and GPi stimulation in both PD and dystonia,^13^, ^57^, ^96^ suggesting that DBS can exert an akinetic effect on speech motor set points analogous to limb or gait hypokinesia.^158^, ^159^ Stuttering‐like disfluencies, whether de novo or representing reemergence of childhood stuttering, are partially reversible with stimulation adjustment. Precise vocal timing in humans requires accurate cortical control in the ventrolateral prefrontal cortex,^160^, ^161^ where the intricate coordination of breathing, articulation, and laryngeal control is enabled through the interaction with the subcortical network. This implicates disrupted timing or initiation within basal ganglia loops, potentially modulated by cerebellar coupling.

Progressive deterioration of speech function over years, particularly in PD, is consistent with maladaptive neuroplasticity superimposed on disease‐specific pathophysiology.^2^, ^3^, ^6^, ^57^, ^59^, ^162^ Acute stimulation effects appear to be gradually overtaken by compensatory network reorganization that may reinforce dysfunctional motor patterns. This interpretation is supported by the limited efficacy of behavioral speech therapy (eg, LSVT LOUD) in post‐DBS patients,^65^, ^114^ in contrast with its established benefit in medically managed PD. This discrepancy suggests that DBS may alter the neural substrates supporting speech motor learning, such as basal ganglia–cortical loops involved in sensorimotor adaptation and reinforcement of learned motor patterns.

Hemispheric lateralization adds a further layer of complexity. Left‐sided stimulation more frequently impaired prosody and articulation than right‐sided stimulation across multiple studies, consistent with the left‐hemisphere dominance of speech‐language networks.^2^, ^7^, ^44^, ^52^, ^57^, ^112^, ^113^ Therefore, in bilateral stimulation, reducing the intensity on the left side may be a reasonable approach when attempting to minimize speech‐related side effects.

As shown in our meta‐analysis, medication state also influences speech outcomes in PD. DBS‐ON/Med‐*on* often shows greater speech impairments than DBS‐ON/Med‐*off*. One possible explanation is that stimulation may inhibit levodopa (l‐dopa) effects by activating pallidal outflow fibers ventral to GPi^163^, ^164^ or dorsal to STN.^165^ In some patients, dyskinesias related to interaction of STN‐DBS and l‐dopa may contribute to speech deterioration.^166^, ^167^ Postoperative reduction in dopaminergic medication may also affect speech outcomes over time. However, the available data do not allow a direct causal link between medication reduction and longitudinal speech decline.

In summary, DBS‐related dysarthria may reflect the interaction of tract‐mediated effects, connectivity‐driven alterations within target nuclei, and longer‐term plasticity, with disease‐specific pathophysiology, hemispheric lateralization, and medication state acting as key modulating factors. Recognizing these phenotypic‐mechanistic alignments may enable a pragmatic programming methodology.

### Clinical Implications for Speech‐Conscious Programming

**FIG. 4:**
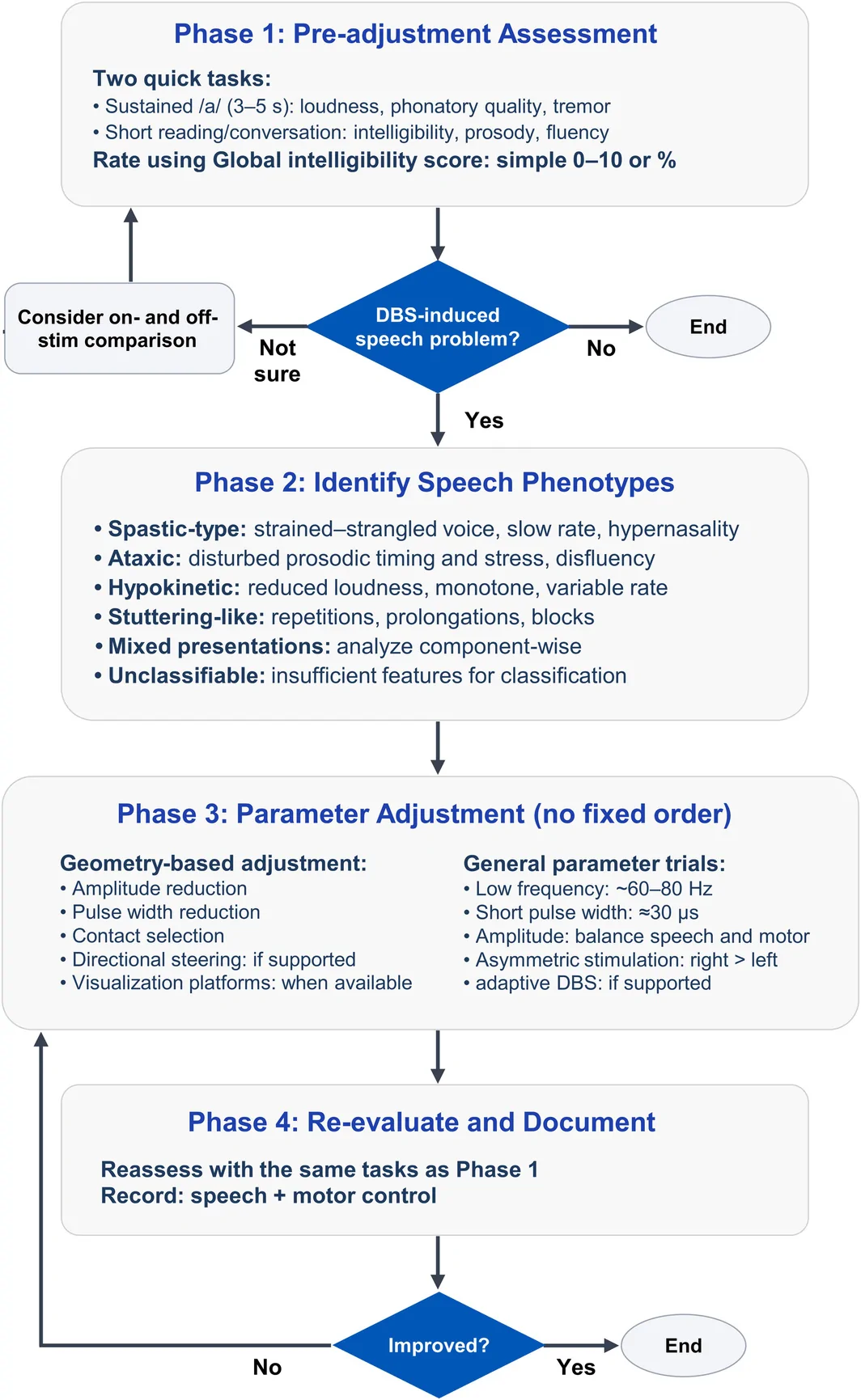
Speech‐conscious deep brain stimulation (DBS) programming workflow. Overview of a four‐phase, iterative workflow for speech‐conscious DBS programming. Patient‐ or caregiver‐reported speech concerns typically serve as the trigger for initiating this evaluation. Phase 1 consists of brief screening using sustained phonation and connected speech to identify clinically relevant speech changes. Phase 2 involves rapid attribution of speech impairment to stimulation through phenotype recognition. Phase 3 applies targeted programming adjustments to balance motor benefit and speech preservation. Phase 4 emphasizes reassessment and documentation, with repetition of the workflow as needed during follow‐up. Because in‐clinic assessments capture only the acute effects of stimulation adjustments and speech changes may emerge over days to weeks, patient‐reported outcome measures (eg, Voice Handicap Index) are recommended to complement clinical evaluation across programming cycles. [Color figure can be viewed at wileyonlinelibrary.com]

Given the frequency and functional impact of DBS‐related speech impairments, structured management approaches have been suggested.^168^, ^169^, ^170^, ^171^ We propose an updated, systematic framework that integrates speech‐conscious evaluation and programming with motor optimization, informed by advances in speech pathophysiology and DBS technology (Fig. 4).

Although structured, scheduled speech evaluation represents the ideal—as outlined in the subsequent section on comprehensive assessment—time constraints during routine programming sessions often limit its feasibility. In practice, patient‐ or caregiver‐reported speech concerns serve as the most practical trigger for initiating detailed assessment. It should also be noted that in‐clinic assessments capture only the acute effects of stimulation adjustments, whereas speech changes may emerge gradually over days to weeks. Accordingly, incorporating PROs such as the VHI before and after stimulation adjustments can complement in‐clinic observations, providing longitudinal, quantitative tracking of communication impact with minimal additional burden on clinicians.

When speech concerns are identified, two standardized tasks provide an efficient starting point: (1) sustained vowel phonation to evaluate vocal intensity, quality, and tremor; and (2) a brief reading and/or conversational sample to assess intelligibility and prosody. A global intelligibility rating (0–10 scale or %) provides sufficient sensitivity for longitudinal tracking and requires minimal clinic time.^172^, ^173^

Identifying the predominant dysarthria phenotype—spastic‐type/ataxic (suggesting tract involvement) versus hypokinetic/stuttering‐like (suggesting network‐level changes)—helps triage next steps and set expectations, particularly in mixed presentations. Brief state comparisons further sharpen attribution: rapid DBS‐OFF sampling (~1 minute) can demonstrate stimulation‐linked components when intelligibility improves promptly, while unilateral testing can isolate hemispheric contributions. Documenting these quick assessments alongside the global intelligibility rating provides a pragmatic pathway to mechanism‐informed adjustments.

No definitive hierarchy exists regarding the optimal sequence of parameter adjustments. Therefore, the following strategies can be explored in a flexible, nonsequential manner. However, when clinical features suggest tract involvement, such as spastic or ataxic speech patterns, it is reasonable to begin with geometric adjustments. This includes modifying contact selection (using directional steering when available), amplitude, or pulse width to redirect current away from corticobulbar and cerebellothalamic pathways. Visualization platforms that localize active contacts relative to patient‐specific tracts can further support decision‐making.

Directional stimulation raises the threshold for stimulation‐induced adverse effects, including dysarthria, compared with omnidirectional stimulation.^154^, ^155^, ^156^, ^157^ Across studies, dysarthria is most commonly associated with lateral current spread toward corticobulbar tracts adjacent to the STN. Directional configurations allow steering of stimulation away from these pathways, thereby expanding the therapeutic window.

Lower frequencies (~60–80 Hz) can stabilize prosody and fluency but may compromise motor control. Shorter pulse widths (~30 μs) may widen the therapeutic window by limiting recruitment of large‐diameter fibers, particularly with capsular proximity.^174^ By contrast, hypokinetic and stuttering‐like presentations lack standardized protocols and often require systematic parameter exploration with unpredictable outcomes. Adaptive DBS guided by neural biomarkers (eg, beta‐band activity) offers a complementary approach by down‐regulating stimulation during Med‐*on* states, potentially minimizing dysarthria while preserving motor benefits. When motor–speech trade‐offs emerge, prioritizing left‐sided parameter reductions is reasonable given hemispheric lateralization.

### Comprehensive Assessment and Longitudinal Management

When resources permit, structured, scheduled evaluation enhances individualized care and enables earlier detection of speech decline.^2^, ^3^, ^6^, ^57^, ^59^, ^162^ A preoperative baseline assessment is essential to anchor interpretation of postoperative change and to help identify patients at higher risk of speech deterioration.

In addition to a global intelligibility measure, comprehensive assessment should include: (1) clinician‐based perceptual evaluations using validated tools (eg, GRBAS, multidimensional dysarthria profiles) to characterize voice quality, resonance, articulation, and prosody; (2) multidimensional acoustic analyses derived from standard speech recordings to provide objective and reproducible metrics of speech performance; and (3) PROs (eg, VHI) to capture the real‐world impact of speech impairment on daily communication and quality of life, which clinicians often underestimate. Recording procedures and task selection should follow standardized protocols to ensure comparability across visits and centers.^175^ Together, integrating these complementary assessment domains within a standardized framework can support longitudinal tracking of speech trajectories and facilitate cross‐center comparisons, advancing both clinical care and research.

Effective longitudinal management benefits from interdisciplinary collaboration among speech‐language pathologists, neurologists, and programmers, particularly when motor–speech trade‐offs require shared decision‐making with patients and caregivers.^176^ Although this review focused on motor speech outcomes, post‐DBS changes in cognitive and neuropsychiatric domains, including verbal fluency decline, apathy, and anxiety, may additionally impair speech communication and should be considered in the holistic management of these patients.

Evidence for speech therapy in the DBS context remains limited.^65^, ^114^ Structured perioperative rehabilitation, including programs such as LSVT LOUD, may help strengthen speech motor control and mitigate postoperative decline, a hypothesis that warrants prospective testing.

### Methodological Limitations

The current evidence base is constrained by substantial methodological heterogeneity and design weaknesses. Speech/voice assessments are highly variable, particularly in acoustic studies reporting dozens of metrics across disparate tasks and nonstandardized recording/processing. Technical reporting is often incomplete, and essential data on patient demographics, stimulation settings, coordinates, and medication states are frequently missing or inconsistently described. Outcome definitions vary across studies, and commonly used scales such as UPDRS‐III item 18 and the BFMDRS scale provide only nonspecific assessments of speech‐related function. Consequently, interpretation of findings from these measures requires caution, because they limit inferences about stimulation‐related speech effects. PROs addressing speech and communication remain underused. Most studies were small, single‐center, cross‐sectional series with limited randomization, blinding, or control groups, and many conducted multiple comparisons without correction while withholding raw audio data for replication. Collectively, these issues hinder comparability, inflate between‐study variance, dilute effect estimates, limit generalizability, and obscure clinicoradiological correlations, such that meta‐analysis was feasible in only two contexts.

### Future Directions

Progress toward speech‐preserving neuromodulation requires coordinated advances across methodological, mechanistic, and therapeutic domains. Harmonized protocols should emphasize connected speech tasks integrating perceptual ratings, acoustic analysis, and PROs under standardized conditions. Complete reporting of baseline demographics and DBS targeting/stimulation parameters is essential. Imaging data on how DBS modulates the neural control of speech remain scarce.^73^, ^111^, ^177^, ^178^, ^179^ In this context, standardized acoustic measures provide an accessible, objective tool for characterization of speech changes and help guide future mechanistic research when integrated with clinical and neuroanatomical data.

Building prospective, standardized longitudinal datasets—linking perceptual, acoustic, and PRO measures to contact topology and stimulation parameters—will be essential to refining programming strategies, benchmarking outcomes, and clarifying long‐term speech trajectories under DBS. In parallel, biomarker‐driven adaptive DBS warrants focused development.^180^ Together, these advances would shift the field toward mechanistically informed strategies that systematically integrate communication outcomes into neuromodulation care.

## Conclusion

Across disorders, DBS reliably improves motor symptoms but may adversely affect speech, particularly connected speech intelligibility. In PD, meta‐analytic evidence indicates that routine STN‐DBS is associated with poorer speech intelligibility compared with best medical therapy, whereas in ET, suppression of voice tremor is offset by an increased risk of dysarthria, especially with bilateral Vim stimulation. Speech outcomes in dystonia are more variable.

Importantly, speech deterioration is neither universal nor inevitable. Converging evidence suggests that DBS‐related speech changes arise from multiple interacting mechanisms, including current spread to specific fiber tracts, disruption of speech networks, and longer‐term maladaptive plasticity. These effects are further shaped by disease‐specific factors, hemispheric lateralization, and medication state.

We propose a pragmatic, speech‐conscious programming approach that integrates brief standardized speech evaluations with phenotype‐informed parameter adjustments. With improved mechanistic understanding and systematic incorporation of speech outcomes into DBS programming and follow‐up, it should be possible to better preserve everyday communication while maintaining motor benefit.

## Author Roles

(1) Research Project: A. Concept and Design, B. Data Acquisition, C. Data Analysis; (2) Statistical Analysis: A. Design, B. Execution, C. Review and Critique; (3) Manuscript Preparation: A. Writing of the First Draft, B. Review and Critique; (4) A. Supervision, B. Funding Acquisition.

E.T.: 1A, 1B, 1C, 2A, 2B, 2C, 3A, 3B, 4A, 4B.

T.T.: 1A, 1B, 1C, 2A, 2B, 2C, 3A, 3B, 4A, 4B.

M.T.B.: 1A, 1B, 2A, 3B, 4A, 4B.

M.S.: 1B, 1C, 3B, 4B.

J.B.‐K.: 1C, 2C, 3B, 4B.

H.J.: 1C, 3B.

G.A.: 1C, 3B.

N.B.: 1C, 3B.

M.D.: 1C, 3B.

P. Krýže: 1B, 1C, 3B, 4B.

J.R.N.: 1C, 3B.

A.E.L.W.: 1C, 3B.

S.R.: 1C, 3B, 4A, 4B.

J.R.: 1B, 1C, 2C,3B, 4B.

P. Krack: 1A, 2C, 3B, 4A, 4B.

K.S.: 1A, 1B, 2A, 3B, 4A, 4B.

## Financial Disclosures and Conflicts of Interest

Author disclosures are available in the Supporting Information.

## Supporting information


**Data S1.** Supporting Information.
**Note S1**. Search terms and data extraction.
**Note S2**. Additional details for the meta‐analyses.
**Table S1A.** Quality review and bias assessment for the studies on deep brain stimulation (DBS) effects on speech for patients with PD.
**Table S1B.** Quality review and bias assessment form for the studies on deep brain stimulation (DBS) effects on speech in patients with ET.
**Table S1C.** Quality review and Bias assessment Form for the studies on deep brain stimulation (DBS) effects on speech in patients with dystonia.
**Table S2.** Acoustic measures (n = 12) and variables (n = 63) reported in the literature of deep brain stimulation (DBS) speech studies.
**Table S3.** Study characteristics.
**Table S4.** Study‐level random‐effects estimates of speech intelligibility.
**Table S5.** Fixed‐effects vs Random‐effects model comparison.
**Table S6.** Leave‐one‐out sensitivity analysis.
**Table S7.** Sensitivity analysis of assumed pre–post correlation (r).
**Table S8.** Study characteristics.
**Table S9.** Study and effect size counts by interval.
**Table S10.** Pooled Δ values by follow‐up interval.
**Table S11.** Publication bias (Egger's test).
**Figure S1.** PRISMA flowchart.
**Figure S2.** Funnel plot of included studies comparing subthalamic nucleus‐deep brain stimulation (STN‐DBS) with best medical therapy for speech intelligibility. Each dot represents an individual study (effect size plotted against its standard error). The distribution appears symmetrical around the pooled effect, and no evidence of small‐study effects was observed. Egger's regression test for funnel plot asymmetry was non‐significant (*z* = −1.35, *P* = 0.18).
**Figure S3.** Funnel plots for assessment of publication bias. (A) Funnel plot of all extracted effect sizes (Δ from baseline) for Unified Parkinson's Disease Rating Scale Part III (UPDRS‐III) item 18. Each dot represents an individual effect size. No significant asymmetry was detected by Egger's regression test (*P* = 0.28). (B) Funnel plot of study‐level pooled Δ values for UPDRS‐III item 18. Each dot represents the aggregated effect within a single study. Egger's regression test approached significance (*P* = 0.054), suggesting that overt publication bias was not confirmed, though the possibility of selective reporting could not be entirely excluded.
**Figure S4.** Due to substantial heterogeneity in tasks, measurement procedures, and reporting formats, statistical meta‐analysis was not possible. We therefore generated a preliminary visual summary using category‐based spider plots. Acoustic variables were grouped into 12 categories and scored as +1, 0, or −1 based on reported effects, with category scores representing mean values across measures. Black and blue markers indicate cross‐sectional and longitudinal outcomes, respectively, and the absence of a marker reflects the lack of data for that category. In panel A (bilateral STN‐DBS in Parkinson's disease; 55 studies), cross‐sectional data showed mild improvements in voicing efficiency, tremor, speech rate, and pauses, whereas longitudinal data generally exhibited minimal change; the most negative findings appeared in fluency (cross‐sectional) and articulation/voice‐onset time (VOT) (longitudinal). In panel B (Vim‐DBS in essential tremor; 10 studies), data points were sparse, with the strongest improvements observed in tremor and pitch, while articulation/VOT and diadochokinesis (DDK) outcomes showed clear deterioration. In panel C (globus pallidus internus [GPi]‐DBS in dystonia; 4 studies), one study reported pronounced improvements in voice quality and intonation, speech rate showed mild benefits, and fluency and pause measures showed slight worsening.

## Data Availability

The data that support the findings of this study are available from the corresponding author upon reasonable request.
