# Supplementary material for: Speech and Deep Brain Stimulation in Parkinson's Disease, Essential Tremor, and Dystonia: A Systematic Review and Meta‐analysis
Source: Mov Disord. 2026 Apr 30;41(8):1928–78. doi: 10.1002/mds.70322 (PMC13518292; doi:10.1002/mds.70322)
Supplement: Supplementary file 1 [file MDS-41-1928-s001.docx]

Supplementary Note 1: search terms and data extraction

**Databases:** Embase Classic+Embase <1947 to 2024 August 30> / APA PsycInfo <1806 to August 2024 Week 5> / Ovid MEDLINE(R) ALL <1946 to August 29, 2024>

| **#** | **Query** | **Results from 2 Sep 2024** |
| --- | --- | --- |
| 1 | exp Parkinson disease/ or exp Parkinson's Disease/ | 326,124 |
| 2 | parkinson*.mp. | 493,353 |
| 3 | spasmodic dysphonia/ | 1,002 |
| 4 | (laryngeal dystonia or spasmodic dysphonia or spastic dysphonia).mp. | 3,090 |
| 5 | exp Dystonia/ or dystonia.mp. | 78,898 |
| 6 | exp Dysphonia/ or dysphonia.mp. | 25,500 |
| 7 | exp tremor/ or exp Essential Tremor/ | 84,691 |
| 8 | (tremor* or tremulous* or trembl* or quiver*).mp. | 125,147 |
| 9 | or/1-8 | 657,084 |
| 10 | exp speech/ or exp voice/ or exp oral communication/ | 275,715 |
| 11 | (speech or spoken or speak or vocal* or phonation or voice or oral).mp. | 3,651,282 |
| 12 | or/10-11 | 3,657,849 |
| 13 | brain depth stimulation/ or deep brain stimulation/ | 73,266 |
| 14 | (deep brain stimulation or DBS).mp. | 65,348 |
| 15 | (brain adj3 (excit* or stimulat*)).mp. | 132,507 |
| 16 | or/13-15 | 146,186 |
| 17 | 9 and 12 and 16 | 3,889 |

**Supplementary Note 2. Additional details for the meta-analyses.**

1. **Overview and prespecified contexts**

Two quantitative syntheses were prespecified:

DBS vs. control (between-group, longitudinal): Prospective studies directly comparing STN-DBS cohorts with medically treated PD controls using speech intelligibility outcomes.

DBS-only (within-group, longitudinal): Studies reporting UPDRS-III item 18 pre- and post-STN-DBS in PD, separately under Med-on and/or Med-off. Pooled estimates were synthesized within follow-up bins (0.5–1 y, 2–4 y, >5 y). All other data were summarized qualitatively due to heterogeneity in design, targets, and outcomes.

**2. Screening, study selection, and overlap checks**

Screening followed PRISMA (search strategy in Supplementary Note 1 and Supplementary Figure 1). For the two meta-analyses, full texts were reassessed against the prespecified criteria. Potential cohort overlap was checked by study site, recruitment period, and sample descriptors; if overlap was suspected, the largest/most comprehensive article for a given time window was retained.

**3. Outcomes, directionality, and harmonization**

DBS vs. control (intelligibility): Outcomes were coded so that positive Hedges’ g = greater improvement in the DBS group.

DBS-only (UPDRS-III item 18): Scale 0–4, higher = worse. Δ = follow-up − baseline; therefore positive Δ = worsening.

Signs were inverted where necessary to maintain these conventions.

**4. Effect-size computation and data conversions**

Between-group: Standardized mean difference with small-sample correction (Hedges’ g). Change-score SMDs were used when available; otherwise post-test SMDs were used.

Within-group: Raw mean difference Δ.

Imputation of pre–post correlation (within-group): r = 0.50 (primary), with r = 0.30 and r = 0.70 in sensitivity analyses.

**5. Handling of multiple effects within studies**

When a study reported more than one eligible effect within the same follow-up bin, we included the earliest qualifying time point only. No study contributed more than one effect per bin after applying this rule; therefore, no aggregation of effects and no cluster-robust estimation were required.

**6. Meta-analytic model, heterogeneity, and sensitivity analyses**

Random-effects models with REML were used. Heterogeneity was summarized with τ², I², and H². Sensitivity analyses included: (i) leave-one-out (DBS vs. control), (ii) fixed- vs. random-effects comparison, and (iii) alternative pre–post r.

**7. Small-study effects and reporting bias**

Funnel plots and Egger’s regression were used when k ≥ 10. For smaller k, plots are provided without formal testing and interpreted cautiously due to low power.

**8. Statistical software and reproducibility**

All analyses were conducted in R (version 4.4.3). The following packages were used: metafor (version 4.8.0; for meta-analysis and heterogeneity statistics), clubSandwich (version 0.6.1; cluster-robust variance estimation), lmtest (version 0.9-40; coefficient testing), ggplot2 (version 3.5.1; figures), dplyr (version 1.1.4), and stringr (version 1.5.1; data wrangling).

**Supplementary Table 1A**: Quality Review and Bias assessment for the studies on DBS effects on speech for patients with PD.

| Author (year) | Selection bias | | | | Confounding bias | | | | | | | | Information bias | | | Selection bias | Confounding bias | Information bias | Total points |  |
| --- | --- | --- | --- | --- | --- | --- | --- | --- | --- | --- | --- | --- | --- | --- | --- | --- | --- | --- | --- | --- |
|  | Type of cohort | Recruitment | Reported on inclusion criteria | Reported on diagnostic criteria | Reported on motor function | Reported on cognitive function | Reported on medication | Reported on disease duration | Reported on age | Reported on pre-op speech data | Reported on speech data with DBS | Reported on electrode positions | Speech assessment | Type of controls | Follow up period | ＞C | ＞B | ＞C |  |  |
| Merola 2011 | A | A | A | A | A | A | A | A | A | A | A | C | A | C | A | 4 | 7 | 2 | 13 |  |
| Ostergaard 2006 | A | A | A | A | A | A | A | A | A | A | A | C | A | C | A | 4 | 7 | 2 | 13 |  |
| Paek 2011 | A | A | A | A | A | B | A | A | A | A | A | A | A | C | A | 4 | 7 | 2 | 13 |  |
| Tsuboi 2017 | A | A | A | A | A | A | A | A | A | B | A | B | A | A | A | 4 | 6 | 3 | 13 |  |
| Aviles-Olmos 2014 | A | A | A | A | A | A | A | A | A | A | B | B | A | C | A | 4 | 6 | 2 | 12 |  |
| Kluin 2022 | A | A | A | A | A | B | A | A | A | A | A | B | A | C | A | 4 | 6 | 2 | 12 |  |
| Krack 2003 | A | A | A | A | A | A | A | A | A | A | B | C | A | C | A | 4 | 6 | 2 | 12 |  |
| Paek 2008 | A | C | A | A | A | A | A | A | A | A | A | B | A | C | A | 3 | 7 | 2 | 12 |  |
| Rousseaux 2004 | A | C | A | A | A | A | A | A | A | A | A | B | A | C | A | 3 | 7 | 2 | 12 |  |
| Tanaka 2020 | A | A | A | A | A | A | A | A | A | B | A | C | A | C | A | 4 | 6 | 2 | 12 |  |
| Tsuboi 2020 | C | A | A | A | A | A | A | A | A | A | B | C | A | A | A | 3 | 6 | 3 | 12 |  |
| Abeyesekera 2019 | A | C | A | A | A | A | A | A | A | C | A | B | A | C | A | 3 | 6 | 2 | 11 |  |
| Fasano 2010 | A | A | A | A | A | A | A | A | A | B | B | C | A | C | A | 4 | 5 | 2 | 11 |  |
| Johansson 2014 | A | A | A | A | A | C | A | A | A | B | A | C | A | C | A | 4 | 5 | 2 | 11 |  |
| Phokaewvarangkul 2019 | B | A | A | A | A | B | A | A | A | C | A | B | A | A | C | 4 | 5 | 2 | 11 |  |
| Pinto 2023 | A | A | A | A | B | C | B | A | A | A | A | C | A | A | A | 4 | 4 | 3 | 11 |  |
| Tandra 2020 | A | C | A | A | A | A | C | A | A | A | A | C | A | C | A | 3 | 6 | 2 | 11 |  |
| Tripoliti 2006 | A | A | A | A | A | C | C | C | A | A | A | C | A | A | A | 4 | 4 | 3 | 11 |  |
| Li 2015 | C | A | A | A | A | B | A | A | A | A | B | C | A | C | A | 3 | 5 | 2 | 10 |  |
| Lundgren 2011 | A | A | A | A | A | C | C | A | A | B | A | C | A | C | A | 4 | 4 | 2 | 10 |  |
| Martel Sauvageau 2015 | A | A | C | C | A | A | A | A | A | A | B | C | A | C | A | 2 | 6 | 2 | 10 |  |
| Svihlik 2022 | B | C | A | A | A | A | B | A | A | C | A | C | A | B | C | 3 | 5 | 2 | 10 |  |
| Tanaka 2015 | B | A | A | A | B | A | B | A | A | C | A | C | A | A | C | 4 | 4 | 2 | 10 |  |
| Thies 2023 | A | C | C | C | A | A | A | A | A | B | A | C | A | B | A | 1 | 6 | 3 | 10 |  |
| Tornqvist 2005 | B | A | A | A | A | C | A | A | A | B | A | B | A | C | C | 4 | 5 | 1 | 10 |  |
| Tripoliti 2011 | A | A | C | C | B | C | A | A | A | A | A | B | A | A | A | 2 | 5 | 3 | 10 |  |
| Tsuboi 2015 | B | C | A | A | B | A | B | A | A | B | A | A | A | A | C | 3 | 5 | 2 | 10 |  |
| Wang 2003 | A | A | A | A | A | B | C | A | A | B | A | C | A | C | A | 4 | 4 | 2 | 10 |  |
| Wang 2006 | A | C | A | A | A | A | C | A | A | B | A | C | A | C | A | 3 | 5 | 2 | 10 |  |
| Catalano Chiuvé 2022 | A | C | C | C | A | A | A | A | A | C | C | C | A | A | A | 1 | 5 | 3 | 9 |  |
| Dromey 2000 | A | A | C | C | A | C | A | C | A | A | A | C | A | C | A | 2 | 5 | 2 | 9 |  |
| Fabbri 2019 | A | C | C | C | B | A | A | A | A | C | A | C | A | A | A | 1 | 5 | 3 | 9 |  |
| Gessani 2023 | C | C | C | C | A | A | A | A | A | A | A | C | A | C | A | 0 | 7 | 2 | 9 |  |
| Hariz 2008 | C | C | A | A | A | B | A | A | A | C | A | B | A | C | A | 2 | 5 | 2 | 9 |  |
| Jergas 2024 | B | A | A | A | B | A |  | C | A | C | A | A | A | C | C | 4 | 4 | 1 | 9 |  |
| Putzer 2008 | B | A | A | A | A | C | C | C | A | C | A | C | A | B | C | 4 | 3 | 2 | 9 |  |
| Rodriguez-Oroz 2005 | A | C | C | C | A | C | A | A | A | A | A | C | A | C | A | 1 | 6 | 2 | 9 |  |
| Sandstrom 2015 | A | C | C | C | C | C | A | A | A | A | A | C | A | B | A | 1 | 5 | 3 | 9 |  |
| Santens 2003 | B | C | A | A | A | C | A | A | A | C | A | B | A | C | C | 3 | 5 | 1 | 9 |  |
| Tanaka 2016 | B | C | A | A | B | A | B | A | A | C | A | C | A | A | C | 3 | 4 | 2 | 9 |  |
| Tanaka 2021 | B | C | A | A | B | A | B | A | A | C | A | C | A | A | C | 3 | 4 | 2 | 9 |  |
| Tripoliti 2011 | A | A | A | A | C | C | C | A | A | C | B | C | A | A | A | 4 | 2 | 3 | 9 |  |
| Tsuboi 2015 | B | C | A | A | B | A | B | A | A | B | A | B | A | A | C | 3 | 4 | 2 | 9 |  |
| Xie 2011 | B | C | A | C | A | C | C | A | A | A | A | C | A | B | C | 2 | 5 | 2 | 9 |  |
| Zibetti 2016 | C | A | C | C | A | C | A | A | A | A | A | B | A | C | A | 1 | 6 | 2 | 9 |  |
| Behroozmand 2019 | B | C | A | A | A | C | B | A | A | C | A | C | A | C | C | 3 | 4 | 1 | 8 |  |
| Bobin 2024 | B | C | A | C | B | C | A | A | A | C | A | A | A | C | C | 2 | 5 | 1 | 8 |  |
| Chiu 2020 | C | C | A | A | A | C | A | A | A | B | B | C | A | C | A | 2 | 4 | 2 | 8 |  |
| El Ouadih 2023 | C | A | C | C | A | C | A | A | A | A | B | C | A | C | A | 1 | 5 | 2 | 8 |  |
| Fabbri 2021 | B | A | A | A | B | A | B | A | A | B | B | B | A | C | C | 4 | 3 | 1 | 8 |  |
| Frost 2010 | A | C | A | A | A | C | C | C | A | B | B | C | A | A | A | 3 | 2 | 3 | 8 |  |
| Gentil 1999 | B | C | A | A | B | C | C | A | A | C | A | C | A | B | C | 3 | 3 | 2 | 8 |  |
| Hammer 2011 | B | C | A | A | A | C | C | A | A | C | A | C | A | C | C | 3 | 4 | 1 | 8 |  |
| Jorge 2020 | C | C | C | A | A | C | A | C | A | A | B | A | A | C | A | 1 | 5 | 2 | 8 |  |
| Karl 2020 | A | C | A | A | B | C | A | A | A | C | B | C | A | C | A | 3 | 3 | 2 | 8 |  |
| Knowles 2018 | A | C | A | C | A | B | A | A | A | B | B | C | A | C | A | 2 | 4 | 2 | 8 |  |
| Knowles 2021 | B | C | A | A | C | A | B | A | A | C | C | C | A | A | C | 3 | 3 | 2 | 8 |  |
| Kryze 2021 | B | C | A | A | B | A | C | A | A | C | B | C | A | B | C | 3 | 3 | 2 | 8 |  |
| Lange 2023 | B | C | A | A | C | C | B | A | A | B | A | A | A | C | C | 3 | 4 | 1 | 8 |  |
| Levy 2023 | B | C | C | C | A | A | A | A | A | B | A | B | A | C | C | 1 | 6 | 1 | 8 |  |
| Martel Sauvageau 2014 | B | C | A | A | B | A | C | A | A | C | A | C | A | C | C | 3 | 4 | 1 | 8 |  |
| Martel Sauvageau 2017 | B | C | A | A | C | C | B | A | A | C | A | C | A | B | C | 3 | 3 | 2 | 8 |  |
| Maruska 2000 | A | C | A | A | B | C | C | A | A | C | A | C | A | C | A | 3 | 3 | 2 | 8 |  |
| Mate 2012 | A | A | A | C | A | C | C | A | A | C | B | C | A | C | A | 3 | 3 | 2 | 8 |  |
| Pinto 2003 | B | C | A | A | A | C | B | A | A | C | A | C | A | C | C | 3 | 4 | 1 | 8 |  |
| Pinto 2014 | A | C | A | C | C | C | B | A | A | A | A | C | A | C | A | 2 | 4 | 2 | 8 |  |
| Shahidi 2017 | C | C | A | C | A | C | A | A | A | A | A | B | C | C | A | 1 | 6 | 1 | 8 |  |
| Sidiropoulos 2013 | A | C | A | A | A | C | C | A | A | B | B | C | A | C | A | 3 | 3 | 2 | 8 |  |
| Sidtis 2012 | B | C | A | A | C | C | C | A | A | C | A | C | A | A | C | 3 | 3 | 2 | 8 |  |
| Sidtis 2016 | B | C | A | A | C | C | C | A | A | C | A | C | A | B | C | 3 | 3 | 2 | 8 |  |
| Skodda 2014 | B | C | A | A | B | C | C | A | A | C | A | C | A | B | C | 3 | 3 | 2 | 8 |  |
| Tripoliti 2008 | B | A | A | A | B | C | C | A | A | A | B | B | A | C | C | 4 | 3 | 1 | 8 |  |
| Tripoliti 2014 | A | A | C | C | B | C | A | A | A | A | B | B | A | C | A | 2 | 4 | 2 | 8 |  |
| Valalik 2011 | B | C | A | A | C | C | C | A | A | C | A | B | A | A | C | 3 | 3 | 2 | 8 |  |
| Ahn 2014 | B | C | A | A | C | B | C | A | A | C | A | C | A | C | C | 3 | 3 | 1 | 7 |  |
| Atkinson-Clement 2017 | B | C | C | A | A | B | C | A | A | C | A | C | A | C | C | 2 | 4 | 1 | 7 |  |
| Cavallieri 2023 | C | A | C | A | B | C | B | A | A | C | A | C | A | C | A | 2 | 3 | 2 | 7 |  |
| D'Alatri 2008 | B | A | A | C | B | B | C | A | A | C | A | C | A | C | C | 3 | 3 | 1 | 7 |  |
| Dayal 2018 | B | A | C | C | A | C | C | A | A | A | B | B | A | C | C | 2 | 4 | 1 | 7 |  |
| Duez 2012 | B | C | A | C | B | C | C | A | A | C | A | C | A | B | C | 2 | 3 | 2 | 7 |  |
| Fenoy 2017 | B | C | C | A | B | B | B | A | A | C | A | A | A | C | C | 2 | 4 | 1 | 7 |  |
| Gentil 2000 | B | C | C | A | B | C | C | A | A | C | A | C | A | B | C | 2 | 3 | 2 | 7 |  |
| Gentil 2003 | B | C | A | A | B | C | C | A | A | C | A | C | A | C | C | 3 | 3 | 1 | 7 |  |
| Hammer 2010 | B | C | C | A | A | C | C | A | A | B | A | C | A | C | C | 2 | 4 | 1 | 7 |  |
| Karlsson 2011 | A | A | C | C | C | C | C | A | A | B | A | C | A | C | A | 2 | 3 | 2 | 7 |  |
| Karlsson 2012 | A | A | C | C | B | C | C | A | A | B | A | C | A | C | A | 2 | 3 | 2 | 7 |  |
| Karlsson 2013 | A | A | C | C | B | C | C | A | A | B | A | C | A | C | A | 2 | 3 | 2 | 7 |  |
| Lee 2008 | B | C | A | A | B | C | C | A | A | C | B | C | A | A | C | 3 | 2 | 2 | 7 |  |
| Mahlknecht 2017 | B | C | A | A | C | C | A | A | A | C | B | B | A | C | C | 3 | 3 | 1 | 7 |  |
| Moreau 2011 | B | C | A | A | B | C | C | A | A | C | A | C | A | C | C | 3 | 3 | 1 | 7 |  |
| Petry-Schmelzer 2022 | A | C | A | A | B | C | B | A | A | B | B | C | A | C | A | 3 | 2 | 2 | 7 |  |
| Pinto 2004 | B | C | C | A | B | C | C | A | A | B | A | C | A | B | C | 2 | 3 | 2 | 7 |  |
| Schulz 2012 | B | C | A | A | B | C | C | A | A | C | A | C | A | C | C | 3 | 3 | 1 | 7 |  |
| Sidtis 2012 | B | C | A | A | C | C | C | C | A | C | A | C | A | A | C | 3 | 2 | 2 | 7 |  |
| Sidtis 2021 | B | C | C | A | B | C | B | A | A | C | A | C | A | A | C | 2 | 3 | 2 | 7 |  |
| Skodda 2011 | B | A | C | C | B | C | C | A | A | C | A | C | A | A | C | 2 | 3 | 2 | 7 |  |
| Skodda 2012 | B | C | C | A | B | C | C | A | A | C | A | C | A | B | C | 2 | 3 | 2 | 7 |  |
| Spielman 2011 | A | C | A | A | C | C | C | C | A | C | B | C | A | A | A | 3 | 1 | 3 | 7 |  |
| van Brenk 2024 | B | C | C | C | A | C | B | A | A | A | A | C | A | C | C | 1 | 5 | 1 | 7 |  |
| Yilmaz 2018 | B | C | A | C | A | C | A | C | A | C | A | C | A | C | C | 2 | 4 | 1 | 7 |  |
| Dayal 2020 | A | C | A | C | B | C | B | A | A | B | B | C | A | C | A | 2 | 2 | 2 | 6 |  |
| Eklund 2015 | A | C | C | C | C | C | C | A | A | B | A | C | A | C | A | 1 | 3 | 2 | 6 |  |
| Grover 2019 | B | C | A | A | B | C | C | A | A | B | B | C | A | C | C | 3 | 2 | 1 | 6 |  |
| Haarmann 2022 | B | A | C | C | C | C | C | A | A | C | C | C | B | A | C | 2 | 2 | 2 | 6 |  |
| Hariz 2008 | C | C | C | C | A | C | A | A | A | C | B | C | A | C | A | 0 | 4 | 2 | 6 |  |
| Jones 2010 | B | C | C | C | A | A | C | C | A | C | A | C | A | C | C | 1 | 4 | 1 | 6 |  |
| Kopf 2022 | B | C | C | C | B | B | A | A | A | A | B | C | B | C | C | 1 | 4 | 1 | 6 |  |
| Lizarraga 2022 | B | C | A | A | B | C | B | A | A | C | B | C | A | C | C | 3 | 2 | 1 | 6 |  |
| Pina-Fuentes 2020 | B | A | A | C | B | B | B | A | A | C | B | C | A | C | C | 3 | 2 | 1 | 6 |  |
| Sarac 2020 | A | C | A | A | C | C | B | C | A | C | B | C | A | C | A | 3 | 1 | 2 | 6 |  |
| Sidtis 2020 | B | C | A | A | C | C | C | C | A | C | A | C | A | C | C | 3 | 2 | 1 | 6 |  |
| Van Lancker Sidtis 2010 | B | C | C | A | B | C | C | A | A | C | A | C | A | C | C | 2 | 3 | 1 | 6 |  |
| Vijiaratnam 2021 | C | C | A | A | B | C | B | A | A | C | B | C | A | C | A | 2 | 2 | 2 | 6 |  |
| Wertheimer 2014 | B | C | A | C | C | C | C | A | A | C | C | C | B | A | C | 2 | 2 | 2 | 6 |  |
| Yasar 2022 | A | C | A | C | C | B | C | C | A | B | A | C | A | C | A | 2 | 2 | 2 | 6 |  |
| Zhou 2009 | B | C | C | C | B | C | B | A | A | B | A | C | A | A | C | 1 | 3 | 2 | 6 |  |
| Ahlberg 2011 | B | C | A | A | C | C | C | A | A | C | B | C | C | C | C | 3 | 2 | 0 | 5 |  |
| Atkinson-Clement 2021 | B | C | C | A | B | C | B | C | A | C | A | C | A | C | C | 2 | 2 | 1 | 5 |  |
| Avantaggiato 2023 | B | C | A | C | B | C | A | A | C | C | B | B | A | C | C | 2 | 2 | 1 | 5 |  |
| Dromey 2011 | B | C | C | C | C | C | C | A | A | C | A | C | A | C | C | 1 | 3 | 1 | 5 |  |
| Gentil 2001 | B | C | C | C | B | C | C | A | A | C | A | C | A | C | C | 1 | 3 | 1 | 5 |  |
| Kim 2024 | B | C | C | C | C | C | B | A | A | C | A | C | A | C | C | 1 | 3 | 1 | 5 |  |
| Klostermann 2008 | B | C | A | C | B | B | C | C | A | C | A | B | A | C | C | 2 | 2 | 1 | 5 |  |
| Morello 2020 | B | C | C | A | B | C | B | A | A | C | B | C | A | C | C | 2 | 2 | 1 | 5 |  |
| Onder 2023 | B | C | C | C | B | C | C | A | A | B | B | C | B | A | C | 1 | 2 | 2 | 5 |  |
| Romann 2019 | B | C | C | C | B | C | C | A | A | C | A | C | A | C | C | 1 | 3 | 1 | 5 |  |
| Sidtis 2017 | B | C | C | C | C | C | C | A | A | C | A | C | A | C | C | 1 | 3 | 1 | 5 |  |
| Astrom 2010 | B | C | C | C | B | C | C | C | A | B | B | A | A | C | C | 1 | 2 | 1 | 4 |  |
| Little 2016 | B | C | C | C | A | C | C | C | C | C | A | C | A | C | C | 1 | 2 | 1 | 4 |  |
| Patel 2015 | C | A | A | C | B | C | C | C | A | C | C | C | C | C | C | 2 | 1 | 0 | 3 |  |
| Picillo 2017 | C | C | C | C | A | C | B | A | A | C | C | B | C | C | C | 0 | 3 | 0 | 3 |  |
| A | Prospective cohort | Consecutive | Fully described | Described | Fully (pre- and post-op) | Formally assessed | Fully (pre- and post-op) | Described | Described | Fully (Med-on and -off) | Fully (DBS-on and -off) | Fully (images and cordinates) | Speech-specific quantitative assessment | Patients non-surgically treated | Follow-up |  |  |  |  |  |
| B | Cross sectional |  |  |  | In part (pre- or post-op) | Descriptively noted | In part (pre- or post-op) |  |  | In part (Med-on or -off) | In part (DBS-on or -off) | In part (images or cordinates) | Speech-specific questionnaires | Healthy controls |  |  |  |  |  |  |
| C | Retrospetive cohort | Other/ not reported | Partially reported/Not described | Not described | Not reported | Not reported | Not reported | Not described | Not described | Not reported | Not reported | Not reported | Non-speech specific scales | No control group | No follow-up |  |  |  |  |  |

Supplementary Table 1B: Quality Review and Bias Assessment Form for the studies on DBS effects on speech in patients with ET

| Author (year) | Selection bias | | | | Confounding bias | | | | | | | | Information bias | | | Selection bias | Confounding bias | Information bias | Total points |  |
| --- | --- | --- | --- | --- | --- | --- | --- | --- | --- | --- | --- | --- | --- | --- | --- | --- | --- | --- | --- | --- |
|  | Type of cohort | Recruitment | Reported on inclusion criteria | Reported on diagnostic criteria | Reported on motor function | Reported on cognitive function | Reported on medication | Reported on disease duration | Reported on age | Reported on pre-op speech data | Reported on speech data with DBS | Reported on electrode positions | Speech assessment | Type of controls | Follow up period | ＞C | ＞B | ＞C |  |  |
| Obwegeser 2000 | A | A | A | A | A | C | C | A | A | A | A | C | C | C | A | 4 | 5 | 1 | 10 |  |
| Sun 2024 | A | A | A | A | A | C | C | A | A | B | B | A | A | C | A | 4 | 4 | 2 | 10 |  |
| Becker 2020 | A | C | A | A | A | C | C | A | A | C | B | B | A | B | A | 3 | 3 | 3 | 9 |  |
| Pulzke 2005 | A | A | C | A | A | C | C | A | A | B | A | B | A | C | A | 3 | 4 | 2 | 9 |  |
| Kronenbuerger 2009 | B | C | C | A | A | C | C | A | A | B | A | C | A | A | C | 2 | 4 | 2 | 8 |  |
| Low 2019 | A | A | A | A | A | C | C | C | A | C | C | B | A | C | A | 4 | 2 | 2 | 8 |  |
| Petry-Schmelzer 2021 | B | A | A | A | B | C | C | C | A | C | A | A | A | C | C | 4 | 3 | 1 | 8 |  |
| Ruckart 2023 | C | A | A | A | A | C | C | A | A | B | B | C | A | C | A | 3 | 3 | 2 | 8 |  |
| Sandstrom 2020 | B | A | C | C | B | C | C | A | A | C | A | A | A | B | C | 2 | 4 | 2 | 8 |  |
| Vogel 2015 | B | A | A | C | B | C | C | A | A | C | A | A | A | C | C | 3 | 4 | 1 | 8 |  |
| Avecillas-Chasin 2018 | B | C | A | C | C | C | C | A | A | C | A | A | A | C | C | 2 | 4 | 1 | 7 |  |
| Erickson-DiRenzo 2020 | A | A | C | C | C | C | C | C | A | A | A | B | A | C | A | 2 | 3 | 2 | 7 |  |
| Matsumoto 2016 | A | A | C | C | A | C | C | C | A | C | B | A | A | C | A | 2 | 3 | 2 | 7 |  |
| Mitchell 2019 | C | A | C | C | A | C | C | A | A | C | A | C | A | C | A | 1 | 4 | 2 | 7 |  |
| Mucke 2014 | B | C | C | C | C | C | C | A | A | C | A | A | A | B | C | 1 | 4 | 2 | 7 |  |
| Pahwa 1999 | A | A | C | C | A | C | C | C | A | C | A | C | A | C | A | 2 | 3 | 2 | 7 |  |
| Ruckart 2022 | C | C | A | A | A | C | C | C | A | C | A | B | A | C | A | 2 | 3 | 2 | 7 |  |
| Becker 2017 | B | C | C | C | B | C | C | A | A | C | A | A | A | C | C | 1 | 4 | 1 | 6 |  |
| Carpenter 1998 | B | A | A | C | B | C | C | C | A | C | A | C | A | C | C | 3 | 2 | 1 | 6 |  |
| Mucke 2018 | B | C | C | C | B | C | C | A | A | C | A | B | A | B | C | 1 | 3 | 2 | 6 |  |
| Patel 2024 | A | A | C | C | C | C | C | C | A | C | B | A | A | C | A | 2 | 2 | 2 | 6 |  |
| Barbe 2014 | B | C | C | C | B | C | C | A | A | C | A | B | A | C | C | 1 | 3 | 1 | 5 |  |
| Hagglund 2016 | B | A | C | C | C | C | C | C | A | C | A | C | A | C | C | 2 | 2 | 1 | 5 |  |
| Sandstrom 2019 | B | C | C | C | B | C | C | A | A | C | A | B | A | C | C | 1 | 3 | 1 | 5 |  |
| Sandstrom 2019 | C | C | A | C | A | C | C | C | A | C | A | C | C | C | A | 1 | 3 | 1 | 5 |  |
| Sandstrom 2021 | B | C | C | C | C | C | C | A | A | C | A | B | A | C | C | 1 | 3 | 1 | 5 |  |
| Boogers 2023 | B | C | C | C | B | C | C | A | A | C | B | B | A | C | C | 1 | 2 | 1 | 4 |  |
| Cernera 2024 | B | C | C | C | B | C | C | A | A | C | B | C | A | C | C | 1 | 2 | 1 | 4 |  |
| Erickson-DiRenzo 2020 | B | C | C | A | B | C | C | C | A | B | B | C | A | C | C | 2 | 1 | 1 | 4 |  |
| Kundu 2018 | C | C | C | C | A | C | C | C | A | B | A | B | C | C | A | 0 | 3 | 1 | 4 |  |
| Sandstrom 2018 | B | C | C | C | C | C | C | C | A | C | A | B | A | C | C | 1 | 2 | 1 | 4 |  |
| Taha 1999 | A | A | C | C | B | C | C | C | C | C | C | C | A | C | A | 2 | 0 | 2 | 4 |  |
| A | Prospective cohort | Consecutive | Fully described | Described | Fully (pre- and post-op) | Formally assessed | Fully (pre- and post-op) | Described | Described | Fully (Med-on and -off) | Fully (DBS-on and -off) | Fully (images and cordinates) | Speech-specific quantitative assessment | Patients non-surgically treated | Follow-up |  |  |  |  |  |
| B | Cross sectional |  |  |  | In part (pre- or post-op) | Descriptively noted | In part (pre- or post-op) |  |  | In part (Med-on or -off) | In part (DBS-on or -off) | In part (images or cordinates) | Speech-specific questionnaires | Healthy controls |  |  |  |  |  |  |
| C | Retrospetive cohort | Other/ not reported | Partially reported/Not described | Not described | Not reported | Not reported | Not reported | Not described | Not described | Not reported | Not reported | Not reported | Non-speech specific scales | No control group | No follow-up |  |  |  |  |  |

Supplementary Table 1C: Quality Review and Bias assessment Form for the studies on DBS effects on speech in patients with Dystonia.

| Author (year) | Selection bias | | | | Confounding bias | | | | | | | | Information bias | | | Selection bias | Confounding bias | Information bias | Total points |  |
| --- | --- | --- | --- | --- | --- | --- | --- | --- | --- | --- | --- | --- | --- | --- | --- | --- | --- | --- | --- | --- |
|  | Type of cohort | Recruitment | Reported on inclusion criteria | Reported on diagnostic criteria | Reported on motor function | Reported on cognitive function | Reported on medication | Reported on disease duration | Reported on age | Reported on pre-op speech data | Reported on speech data with DBS | Reported on electrode positions | Speech assessment | Type of controls | Follow up period | ＞C | ＞B | ＞C |  |  |
| Kupsch 2006 | A | A | A | C | A | A | C | A | A | C | A | B | A | C | A | 3 | 5 | 2 | 10 |  |
| Honey 2021 | A | C | C | C | A | A | C | A | A | C | A | B | A | C | A | 1 | 5 | 2 | 8 |  |
| Pauls 2018 | B | A | A | C | B | B | C | A | A | C | A | A | A | C | C | 3 | 4 | 1 | 8 |  |
| Sobstyl 2017 | A | A | A | C | A | B | C | A | A | A | B | C | C | C | A | 3 | 4 | 1 | 8 |  |
| Evidente 2020 | C | C | C | A | A | C | C | A | A | C | A | A | A | C | C | 1 | 5 | 1 | 7 |  |
| Isaias 2009 | C | A | C | A | A | B | C | A | A | C | B | C | A | C | A | 2 | 3 | 2 | 7 |  |
| Ouyang 2021 | A | A | A | C | A | C | C | A | A | C | C | B | C | C | A | 3 | 3 | 1 | 7 |  |
| Risch 2015 | B | A | C | C | C | C | C | A | A | C | A | C | A | B | C | 2 | 3 | 2 | 7 |  |
| Rusz 2018 | B | C | C | C | A | C | C | A | A | C | A | B | A | B | C | 1 | 4 | 2 | 7 |  |
| Tian 2021 | C | A | C | A | A | C | C | A | A | C | B | B | A | C | A | 2 | 3 | 2 | 7 |  |
| Volkmann 2014 | A | C | A | C | B | A | C | A | A | C | A | C | C | C | A | 2 | 4 | 1 | 7 |  |
| Danielsson 2019 | C | C | A | A | A | C | C | A | A | B | C | C | C | C | A | 2 | 3 | 1 | 6 |  |
| Finger 2020 | C | C | A | C | A | C | C | A | A | B | B | C | A | C | A | 1 | 3 | 2 | 6 |  |
| Reese 2011 | C | C | A | A | A | B | C | A | A | C | B | B | C | C | A | 2 | 3 | 1 | 6 |  |
| Ren 2022 | C | C | A | A | A | C | C | A | A | B | B | B | C | C | A | 2 | 3 | 1 | 6 |  |
| Rusz 2019 | B | C | C | C | B | C | C | A | A | C | A | B | A | B | C | 1 | 3 | 2 | 6 |  |
| Sensi 2009 | C | C | A | C | A | A | C | A | A | B | B | B | C | C | A | 1 | 4 | 1 | 6 |  |
| Tian 2020 | C | C | A | C | A | A | C | A | A | C | B | B | C | C | A | 1 | 4 | 1 | 6 |  |
| Hao 2020 | C | A | C | C | A | C | C | A | A | C | B | C | C | C | A | 1 | 3 | 1 | 5 |  |
| Groen 2010 | C | A | C | A | A | C | C | C | A | C | C | C | C | C | C | 2 | 2 | 0 | 4 |  |
| Limotai 2011 | C | C | C | C | A | C | C | A | A | C | C | B | C | C | A | 0 | 3 | 1 | 4 |  |
| A | Prospective cohort | Consecutive | Fully described | Described | Fully (pre- and post-op) | Formally assessed | Fully (pre- and post-op) | Described | Described | Fully (Med-on and -off) | Fully (DBS-on and -off) | Fully (images and cordinates) | Speech-specific quantitative assessment | Patients non-surgically treated | Follow-up |  |  |  |  |  |
| B | Cross sectional |  |  |  | In part (pre- or post-op) | Descriptively noted | In part (pre- or post-op) |  |  | In part (Med-on or -off) | In part (DBS-on or -off) | In part (images or cordinates) | Speech-specific questionnaires | Healthy controls |  |  |  |  |  |  |
| C | Retrospetive cohort | Other/ not reported | Partially reported/Not described | Not described | Not reported | Not reported | Not reported | Not described | Not described | Not reported | Not reported | Not reported | Non-speech specific scales | No control group | No follow-up |  |  |  |  |  |

Supplementary Figure 1. PRISMA flowchart.

Supplementary Table 2: Acoustic measures (N=12) and variables (N=63) reported in the literature of DBS speech studies

| Pitch | **Mean Fundamental Frequency (F0)**  **Fundamental Frequency SD (F0 SD)**  **Minimum Pitch (f0)**  **Maximum Pitch (f0)**  **Maximum f0 Range (Pitch Range)**  **Vocal pitch compensation (Cents)** |
| --- | --- |
| Loudness | **Mean Voice Intensity (dB or SPL)**  **Voice intensity SD (dB)**  **Minimum Loudness (dB or SPL)**  **Maximum Loudness (dB or SPL)**  **Maximum Intensity Range (Loudness Range)** |
| Voicing efficiency | **Maximum Phonation Duration (MPT)** |
| Voice quality | **Jitter**  **Shimmer**  **Noise-to-Harmonic Ratio (NHR) or HNR or SNR**  **Cepstral Spectral Index of Dysphonia (CSID)**  **Cepstral peak prominence (CPP)**  **CPP SD**  **CPP F0**  **CPP F0 SD**  **Degree of subharmonics**  **Degree of unvoiced segment** (measure of strained-strangled voice)  **Time-delayed state-space recurrence analysis (TDSSRA)**  **Measure based on nonlinear dynamics (D2, RPDE, PPE, DFA etc.)** |
| Tremor | **Rate of F0 modulation (tremor)**  **Extent of F0 modulation (tremor)**  **Rate of amplitude modulation (tremor)**  **Extent of amplitude modulation (tremor)**  **Amplitude Tremor Intensity Index**  **Frequency Tremor Intensity Index**  **Rate of Acoustic Tremor (modulation)**  **Mean amplitude of Acoustic Tremor (modulation)**  **Coefficient of Variation of F0**  **Coefficient of Variability** |
| Intonation | **Frequency variability**  **Amplitude variability** |
| Vowel articulation | **Vowel Articulation Space:**  Corner vowel space (F1/F2 ratio)  **Corner vowel formant values (/a/, /i/, /u/:)** Corner vowel space from F1, F2, and/or F3  **FFT of F1, F2, F3 across corner vowels**  **F2 slope**  **duration of vowels selected for analysis**  **Mean Long-term average spectrum (FFT)**  **Long-term average spectrum (FFT) SD**  **Articulation Acceleration** |
| Articulation (Voice onset time) | **Voice Onset Time (VOT)**  **Voicing-to-syllable ratio**  **Voicing-during-closure** (spectrographic )  **Frication-during-closure** (spectrographic) |
| Speech rate | **Speech Rate (syllables/sec)**  **Mean duration of articulated speech**  **Articulation Rate** |
| Fluency | **% Syllables Stuttered** |
| Fluency measures | **Mean Syllable Duration**  **DDK Rate (Number of syllables per time)**  **DDK Stability/Regularity**  **Mean vowel duration**  **Intensity decay**  **Coefficient of Variation of DDK Rate Period**  (variability of speech rate) |
| Pauses | **Number of Prolonged pauses**  **Number of Pauses Relative to Total Speech Time**  **Mean Silence Time**  **Variability of Silence Length**  **Percentage of Silence in a Speech Sample** |

| Supplementary Table 3. Study characteristics | | | | | | | | |
| --- | --- | --- | --- | --- | --- | --- | --- | --- |
| Study | Group | N | Mean_Pre | SD_Pre | Mean_Post | SD_Post | Mean  Change | SD  Change |
| Pinto_2023_DE | DBS | 94 | 91.66 | 9.55 |  |  | -1.19 | 10.27 |
| Pinto_2023_DE | Control | 51 | 90.45 | 9.94 |  |  | 0.33 | 10.54 |
| Pinto_2023_FR | DBS | 94 | 91.16 | 9.74 |  |  | -4.12 | 11.22 |
| Pinto_2023_FR | Control | 51 | 90.35 | 9.87 |  |  | -1.95 | 10.89 |
| Tripoliti_2011 | DBS | 32 | 77.6 | 15 | 61.7 | 26 |  |  |
| Tripoliti_2011 | Control | 12 | 76.3 | 5.8 | 71.8 | 8.4 |  |  |
| Tsuboi_2017 | DBS | 32 | 87.5 | 15 | 77.5 | 15 |  |  |
| Tsuboi_2017 | Control | 11 | 85 | 12.5 | 82.5 | 10 |  |  |

| Supplementary Table 4. Study-level random-effects estimates of speech intelligibility | | | | |
| --- | --- | --- | --- | --- |
| Study | g | ci_lb | ci_ub | p |
| Pinto_2023_DE | -0.31 | -0.589 | -0.0313 | 0.0293 |
| Pinto_2023_FR | -0.293 | -0.598 | 0.0111 | 0.0589 |
| Tripoliti_2011 | -0.208 | -0.436 | 0.0204 | 0.0743 |
| Tsuboi_2017 | -0.215 | -0.442 | 0.0122 | 0.0636 |

| Supplementary Table 5. Fixed-effects vs Random-effects model comparison | | | | | | |
| --- | --- | --- | --- | --- | --- | --- |
| Model | pooled_g | ci_lb | ci_ub | p | tau2 | I2 |
| Fixed-effects | -0.244 | -0.46 | -0.0284 | 0.0265 |  |  |
| Random-effects (REML) | -0.244 | -0.46 | -0.0284 | 0.0265 | 0 | 0 |

| Supplementary Table 6. Leave-one-out sensitivity analysis | | | | |
| --- | --- | --- | --- | --- |
| Study | pooled_g | ci_lb | ci_ub | p |
| Pinto_2023_DE | -0.31 | -0.589 | -0.0313 | 0.0293 |
| Pinto_2023_FR | -0.293 | -0.598 | 0.0111 | 0.0589 |
| Tripoliti_2011 | -0.208 | -0.436 | 0.0204 | 0.0743 |
| Tsuboi_2017 | -0.215 | -0.442 | 0.0122 | 0.0636 |

| Supplementary Table 7. Sensitivity analysis of assumed pre–post correlation (r) | | | | | | |
| --- | --- | --- | --- | --- | --- | --- |
| r | pooled_g | ci_lb | ci_ub | p | tau2 | I2 |
| 0.3 | -0.23 | -0.446 | -0.0139 | 0.0369 | 0 | 0 |
| 0.5 | -0.244 | -0.46 | -0.0284 | 0.0265 | 0 | 0 |
| 0.7 | -0.269 | -0.486 | -0.0531 | 0.0146 | 3.28E-07 | 0.000591 |

**Supplementary Figure 2.** Funnel plot of included studies comparing STN-DBS with best medical therapy for speech intelligibility. Each dot represents an individual study (effect size plotted against its standard error). The distribution appears symmetrical around the pooled effect, and no evidence of small-study effects was observed. Egger’s regression test for funnel plot asymmetry was non-significant (z = −1.35, p = 0.18).

| Supplementary Table 8. Study characteristics | | | | | | |
| --- | --- | --- | --- | --- | --- | --- |
| Stuties | Followup_months | Med_state | Stim_state | n | mean | sd |
| Fasano_2010 | 0 | Med-off | DBS-off | 20 | 2.2 | 0.7 |
| Fasano_2010 | 12 | Med-off | DBS-on | 20 | 1.7 | 0.9 |
| Fasano_2010 | 36 | Med-off | DBS-on | 20 | 1.9 | 0.8 |
| Fasano_2010 | 60 | Med-off | DBS-on | 20 | 1.9 | 0.9 |
| Fasano_2010 | 96 | Med-off | DBS-on | 20 | 2.3 | 0.9 |
| Fasano_2010 | 0 | Med-on | DBS-off | 20 | 1.3 | 0.7 |
| Fasano_2010 | 12 | Med-on | DBS-on | 20 | 1.4 | 0.9 |
| Fasano_2010 | 36 | Med-on | DBS-on | 20 | 1.9 | 0.9 |
| Fasano_2010 | 60 | Med-on | DBS-on | 20 | 1.7 | 1.1 |
| Fasano_2010 | 96 | Med-on | DBS-on | 20 | 2.2 | 1 |
| Krack_2003 | 0 | Med-off | DBS-off | 49 | 1.9 | 1 |
| Krack_2003 | 12 | Med-off | DBS-on | 43 | 1.3 | 1 |
| Krack_2003 | 36 | Med-off | DBS-on | 42 | 1.8 | 1 |
| Krack_2003 | 60 | Med-off | DBS-on | 42 | 1.9 | 1 |
| Krack_2003 | 0 | Med-on | DBS-off | 49 | 0.8 | 0.6 |
| Krack_2003 | 12 | Med-on | DBS-on | 43 | 0.9 | 0.7 |
| Krack_2003 | 36 | Med-on | DBS-on | 40 | 1.4 | 0.9 |
| Krack_2003 | 60 | Med-on | DBS-on | 39 | 1.8 | 0.7 |
| Østergaard_2006 | 0 | Med-off | DBS-off | 26 | 1.2 | 0.8 |
| Østergaard_2006 | 12 | Med-off | DBS-off | 26 | 1 | 0.8 |
| Østergaard_2006 | 12 | Med-off | DBS-on | 26 | 0.9 | 0.7 |
| Østergaard_2006 | 48 | Med-off | DBS-off | 18 | 1.6 | 1 |
| Østergaard_2006 | 48 | Med-off | DBS-on | 22 | 1.6 | 1 |
| Østergaard_2006 | 0 | Med-on | DBS-off | 26 | 0.7 | 0.8 |
| Østergaard_2006 | 12 | Med-on | DBS-off | 26 | 0.7 | 0.7 |
| Østergaard_2006 | 12 | Med-on | DBS-on | 26 | 0.6 | 0.8 |
| Østergaard_2006 | 48 | Med-on | DBS-off | 17 | 1.6 | 0.8 |
| Østergaard_2006 | 48 | Med-on | DBS-on | 20 | 1.4 | 0.9 |
| Paek_2011 | 0 | Med-off | DBS-off | 57 | 1.5 | 0.7 |
| Paek_2011 | 6 | Med-off | DBS-on | 57 | 1.4 | 0.8 |
| Paek_2011 | 12 | Med-off | DBS-on | 57 | 1.4 | 0.8 |
| Paek_2011 | 0 | Med-on | DBS-off | 57 | 1.2 | 0.7 |
| Paek_2011 | 6 | Med-on | DBS-on | 57 | 1.2 | 0.8 |
| Paek_2011 | 12 | Med-on | DBS-on | 57 | 1.3 | 0.9 |
| Rodriguez-Oroz_2005 | 0 | Med-off | DBS-off | 49 | 1.9 | 1 |
| Rodriguez-Oroz_2005 | 0 | Med-on | DBS-off | 49 | 1.1 | 0.9 |
| Rodriguez-Oroz_2005 | 12 | Med-off | DBS-on | 49 | 1.4 | 0.9 |
| Rodriguez-Oroz_2005 | 12 | Med-on | DBS-on | 49 | 1.2 | 0.9 |
| Rodriguez-Oroz_2005 | 42 | Med-off | DBS-on | 49 | 1.8 | 1 |
| Rodriguez-Oroz_2005 | 42 | Med-on | DBS-on | 49 | 1.6 | 0.9 |
| Rousseaux_2004 | 0 | Med-off | DBS-off | 7 | 2 | 0.58 |
| Rousseaux_2004 | 0 | Med-on | DBS-off | 7 | 1.14 | 0.69 |
| Rousseaux_2004 | 3 | Med-off | DBS-off | 7 | 1.29 | 0.76 |
| Rousseaux_2004 | 3 | Med-on | DBS-off | 7 | 1 | 0.58 |
| Rousseaux_2004 | 3 | Med-off | DBS-on | 7 | 1.29 | 0.95 |
| Rousseaux_2004 | 3 | Med-on | DBS-on | 7 | 1 | 0.58 |
| Tandra_2020 | 0 | Med-off | DBS-off | 40 | 1.55 | 0.8 |
| Tandra_2020 | 6 | Med-off | DBS-on | 40 | 1.15 | 0.06 |
| Tandra_2020 | 0 | Med-on | DBS-off | 40 | 0.22 | 0.4 |
| Tandra_2020 | 6 | Med-on | DBS-on | 40 | 0.2 | 0.4 |
| El Ouadih_2023 | 0 | Med-off | DBS-off | 92 | 0.79 | 0.67 |
| El Ouadih_2023 | 12 | Med-off | DBS-on | 90 | 1.33 | 0.87 |
| El Ouadih_2023 | 0 | Med-on | DBS-off | 91 | 0.51 | 0.63 |
| El Ouadih_2023 | 12 | Med-on | DBS-on | 61 | 1.18 | 0.86 |
| Gessani_2023 | 0 | Med-off | DBS-off | 25 | 1.35 | 0.57 |
| Gessani_2023 | 0 | Med-on | DBS-off | 25 | 0.608 | 0.65 |
| Gessani_2023 | 60 | Med-off | DBS-on | 25 | 1.52 | 0.71 |
| Gessani_2023 | 60 | Med-off | DBS-off | 25 | 1.68 | 0.74 |
| Gessani_2023 | 60 | Med-on | DBS-on | 25 | 1.44 | 0.65 |
| Jorge_2020 | 0 | Med-off | DBS-off | 13 | 1.1 | 0.6 |
| Jorge_2020 | 0 | Med-on | DBS-off | 13 | 1 | 0.7 |
| Jorge_2020 | 7 | Med-on | DBS-on | 5 | 0.5 | 0.6 |
| Li_2015 | 0 | Med-off | DBS-off | 195 | 2.1 | 1.1 |
| Li_2015 | 12 | Med-off | DBS-on | 187 | 1.9 | 1.2 |
| Li_2015 | 36 | Med-off | DBS-on | 170 | 1.8 | 1.3 |
| Li_2015 | 60 | Med-off | DBS-on | 70 | 2 | 1.2 |
| Li_2015 | 0 | Med-on | DBS-off | 195 | 0.9 | 0.5 |
| Li_2015 | 12 | Med-on | DBS-on | 187 | 0.9 | 0.7 |
| Li_2015 | 36 | Med-on | DBS-on | 170 | 1.4 | 0.9 |
| Li_2015 | 60 | Med-on | DBS-on | 70 | 1.9 | 0.8 |
| Shahidi_2017 | 0 | Med-on | DBS-off | 36 | 1.1 | 0.7 |
| Shahidi_2017 | 6 | Med-on | DBS-on | 36 | 1.05 | 0.89 |
| Shahidi_2017 | 72 | Med-on | DBS-on | 14 | 1.9 | 1.07 |

| Supplementary Table 9. Study and effect size counts by interval | | | |
| --- | --- | --- | --- |
| Condition | Interval | n studies | n effect sizes |
| Med-off | 0.5–1 years | 7 | 8 |
| Med-off | 2–4 years | 5 | 5 |
| Med-off | >5 years | 4 | 5 |
| Med-on | 0.5–1 years | 9 | 10 |
| Med-on | 2–4 years | 5 | 5 |
| Med-on | >5 years | 5 | 6 |

| Supplementary Table 10. Pooled Δ values by follow-up interval | | | | | |
| --- | --- | --- | --- | --- | --- |
| Condition | Follow-up interval | Pooled Δ (mean) | SE | 95% CI | k (effect sizes) |
| Med-off | 0.5–1 years | -0.095 | 0.038 | –0.170 to –0.020 | 8 |
| Med-off | 2–4 years | -0.164 | 0.061 | –0.283 to –0.044 | 5 |
| Med-off | >5 years | -0.006 | 0.069 | –0.140 to +0.129 | 5 |
| Med-on | 0.5–1 years | 0.065 | 0.028 | +0.010 to +0.120 | 10 |
| Med-on | 2–4 years | 0.532 | 0.047 | +0.441 to +0.624 | 5 |
| Med-on | >5 years | 0.92 | 0.053 | +0.816 to +1.020 | 6 |

**Supplementary Figure 3:** Funnel plots for assessment of publication bias. (A) Funnel plot of all extracted effect sizes (Δ from baseline) for UPDRS-III item 18. Each dot represents an individual effect size. No significant asymmetry was detected by Egger’s regression test (p = 0.28). (B) Funnel plot of study-level pooled Δ values for UPDRS-III item 18. Each dot represents the aggregated effect within a single study. Egger’s regression test approached significance (p = 0.054), suggesting that overt publication bias was not confirmed, though the possibility of selective reporting could not be entirely excluded.

| Supplementary Table 11. Publication bias (Egger’s test) | | | |
| --- | --- | --- | --- |
| Analysis level | Egger’s test (z) | p-value | Interpretation |
| Effect-size level | -1.09 | 0.28 | No evidence of asymmetry |
| Study-level pooled | -1.93 | 0.054 | Borderline; not significant |

**Supplementary Figure 4:** Due to substantial heterogeneity in tasks, measurement procedures, and reporting formats, statistical meta-analysis was not possible. We therefore generated a preliminary visual summary using category-based spider plots. Acoustic variables were grouped into 12 categories and scored as +1, 0, or –1 based on reported effects, with category scores representing mean values across measures. Black and blue markers indicate cross-sectional and longitudinal outcomes, respectively, and the absence of a marker reflects the lack of data for that category. In panel A (bilateral STN-DBS in Parkinson’s disease; 55 studies), cross-sectional data showed mild improvements in voicing efficiency, tremor, speech rate, and pauses, whereas longitudinal data generally exhibited minimal change; the most negative findings appeared in fluency (cross-sectional) and articulation/VOT (longitudinal). In panel B (Vim-DBS in essential tremor; 10 studies), data points were sparse, with the strongest improvements observed in tremor and pitch, while articulation/VOT and DDK outcomes showed clear deterioration. In panel C (GPi-DBS in dystonia; 4 studies), one study reported pronounced improvements in voice quality and intonation, speech rate showed mild benefits, and fluency and pause measures showed slight worsening.

Abbreviations: DBS, deep brain stimulation; DDK, diadochokinesis; ET, essential tremor; GPi, globus pallidus internus; PD, Parkinson’s disease; STN, subthalamic nucleus; Vim, ventralis intermedius nucleus of the thalamus; VOT, voice onset time.
